# Cost-effectiveness of digital health in primary care: a systematic review

**DOI:** 10.1186/s12913-026-16041-5

**Published:** 2026-11-06

**Authors:** Sandra Stern, Anna Johansson, Evalill Nilsson, Lina Nilsson

**Affiliations:** 1 eHealth Institute, Department of Medicine and Optometry, Linnaeus University, Kalmar, Sweden; 2 Department of Research and Development, Växjö, Region Kronoberg Sweden

**Keywords:** Cost-effectiveness, Health economics, Digital health, E-health, Primary care, Systematic review

## Abstract

**Background:**

Digital health has rapidly expanded, accelerated by the COVID-19 pandemic, and is widely used for consultations, disease management, and efficiency improvements in primary care. While it is considered clinically effective and potentially cost-effective, evidence on economic impact remains limited. Previous reviews have been condition-specific, highlighting the need for a systematic review focused on cost-effectiveness of digital health within primary care.

**Objectives:**

The aim of this systematic review was to summarize and investigate the cost-effectiveness of digital health among adult primary care patients.

**Methods:**

A systematic literature search was conducted using the PICO framework. Studies were included if they involved adult primary care patients (≥18 years), evaluated digital health interventions versus usual care, and reported cost‑effectiveness based on economic evaluations. PubMed, Scopus, and Health Business Elite were searched in January 2025. An updated search was conducted in September 2026. Results were reported in a Preferred Reporting Items for Systematic Reviews and Meta-Analyses (PRISMA) flow diagram. Methodological quality was assessed using the Joanna Briggs Institute Checklist for Economic Evaluations. A narrative synthesis was performed.

**Results:**

The initial search identified 4,091 records and resulted in 47 included studies. The updated search in September 2026 identified an additional 658 records, of which seven additional studies met the eligibility criteria, resulting in a total of 54 included studies. Most were trial‑based full economic evaluations focusing on digital interventions for depression and anxiety, adopting a healthcare perspective. Overall, digital health interventions were frequently associated with favourable economic outcomes, although results varied by intervention, population, economic evaluation method, and time horizon.

**Discussion:**

This review is innovative in its focus on the cost‑effectiveness of digital health interventions in primary care. Unlike previous reviews, it systematically synthesises trial‑ and model‑based economic evaluations. The review advances the field by clarifying the economic evidence base and has practical implications for decision‑makers considering the implementation of digital health in primary care. The review is limited by rapidly evolving digital health terminology, the exclusion of grey literature, and substantial methodological heterogeneity between trial‑based and model‑based economic evaluations. Potential selection bias related to digital literacy may limit generalisability.

**Conclusions:**

Digital health in primary care demonstrated favourable economic outcomes across a broad range of clinical areas, with the majority of studies reporting cost-effective results. However, despite this positive pattern, methodological heterogeneity limits comparability between studies. Greater standardization of economic evaluation methods and reporting is therefore needed to strengthen the evidence base and support implementation decisions.

**Trial Registration:**

PROSPERO registration no. CRD42025644904; [https://www.crd.york.ac.uk/PROSPERO/view/CRD42025644904].

**Supplementary information:**

The online version contains supplementary material available at https://doi.org/10.1186/s12913-026-16041-5.

## Introduction

### Rationale

The rapid development of digital technologies has transformed multiple sectors of society, including healthcare. Technological innovation has driven the development of digital health and enabled new forms of communication, diagnostics, and care delivery [1, 2]. In many public healthcare systems, the adoption of digital health accelerated sharply during the COVID-19 pandemic. Reducing onsite visits through the use of digital health helped limit the spread of infection and provided greater flexibility for both patients and healthcare professionals by enabling synchronous or asynchronous interaction without meeting in person [3, 4]. Since then, digital health has continued to expand [5] and is now used for digital consultations, managing illnesses, supporting wellness, improving efficiency through more accessible, personalized, and data-driven services. The World Health Organization (WHO) defines digital health as *“the field of knowledge and practice associated with the development and use of digital technologies to improve health”* [6].

Digital health is increasingly viewed as an important strategy for addressing the growing pressure on primary care [7, 8] and has been shown to be diagnostically safe, clinically effective, and in some cases cost-effective [9, 10]. However, although digital health is often introduced with the expectation of reducing rising healthcare costs, the existing evidence does not yet allow for firm conclusions [11, 12]. In this study, primary care is defined in line with the World Health Organization as first-contact health care that is accessible, continuous, comprehensive, and coordinated, and that addresses the majority of individuals’ health needs throughout the life course [13].

Health economic evaluations, such as cost-effectiveness analyses, provide important tools for assessing the value of healthcare interventions. These evaluations compare costs and outcomes to support decision-making and guide resource allocation [14]. Despite the increasing implementation of digital health services, many are still introduced without robust evidence of their economic implications [11, 15].

Previous systematic reviews have examined the effects and costs of digital health, but these reviews often focus on specific conditions or narrowly defined interventions, such as nutritional therapy [16] or diabetes management [17]. Few reviews have specifically addressed digital health within primary care settings. Among these are reviews with a broader scope, encompassing the implementation and evaluation of a wide range of digital health solutions, such as monitoring systems, mobile apps and remote patient monitoring [18–21]. Earlier systematic reviews on video communication have indicated cost-effectiveness [22, 23]; however, numerous new studies have been published since then. Given the rapid development of digital health, a focused review of cost-effectiveness in primary care is both timely and relevant. Primary care is typically delivered by general practitioners, family physicians, nurse practitioners, and other primary healthcare professionals [24]. It should be acknowledged that the organization and delivery of primary care may differ across countries.

### Objective

The aim of this systematic review is to summarize and investigate the cost-effectiveness of digital health among adult primary care patients.

### Methods

The objectives, inclusion criteria, and methods for this review were pre-specified and documented in a protocol (PROSPERO registration no. CRD42025644904). The registered study protocol was followed in all substantive aspects. The primary deviation relates to the number of databases included, which was adjusted during the review process to balance feasibility and yield.

The search strategy was developed a priori using the PICO framework (Population, Intervention/Exposure, Comparison, Outcome) to ensure conceptual clarity and comprehensive retrieval of relevant studies. Both controlled vocabulary and free-text terms were used. Controlled vocabulary terms (MeSH) were identified using the Karolinska Institutet MeSH browser, in collaboration with a research librarian, whose contribution is acknowledged. The complete search strategies, including the exact search strings, Boolean operators, and execution details for each database, are provided in Supplementary Appendix 1.

### Eligibility criteria

Papers were eligible for inclusion if they met all of the specified criteria. The eligibility criteria used for study selection are presented in Table 1.

**Table 1:** Eligibility criteria

	Inclusion criteria	Exclusion criteria
Population	Adult primary care patients (≥18 years).	
Intervention	Consultations or therapeutic interventions delivered through digital technologies enabling communication between patients and healthcare professionals in primary care.	Self-monitoring, interventions within secondary care.
Comparator	Usual care defined as onsite visits in primary care.	Studies using waitlist controls as the sole comparator.
Outcomes	Cost-effectiveness. Includes full and partial economic evaluation.	Studies reporting only clinical outcomes.
Study design	Within-trial or model-based health economic evaluations. Original quantitative studies or quantitative results from mixed-methods studies.	Purely qualitative studies. Reviews, editorials, commentaries, and methodological articles.
Language	English and Nordic languages.	
Date	2015 to present.	

### Types of populations

This systematic review includes studies on patients and target groups who receive or are registered to receive medical care, treatment, or advice from a healthcare professional or institution in primary care and who use digital health. Digital health targeting children and young people are subject to distinct regulatory constraints and implementation conditions that would have required separate analytical considerations. To maintain internal consistency and feasibility, the review therefore focused on adult populations. There is no exclusion based on occupational category.

### Types of interventions

The interventions considered in this review include consultations and therapeutic procedures conducted using digital health methods that facilitate interaction between healthcare providers and patients in primary care. Both synchronous and asynchronous communication methods are included. Digital triage without treatment or intervention, purely digital self-care advice and self-monitoring are excluded. The time span from 2015 onwards was chosen because it reflects the rapid technological developments of the past decade and encompasses the years of the COVID-19 pandemic, during which digital health expanded.

### Types of comparators

The comparator in this systematic review is usual care, here defined as in-person visits within primary care. Primary care is characterized by a complex healthcare environment marked by diagnostic diversity, a broad spectrum of patient needs, and interdisciplinary collaboration among various professional groups. Therefore, no exclusions were applied based on diagnosis or professional category.

### Types of outcomes

This review includes both trial-based and model-based economic evaluations. Trial-based economic evaluations are conducted alongside clinical trials and use data collected directly from trial participants [25]. Model-based economic evaluations use decision-analytic models (e.g., Markov models, decision trees) to simulate costs and outcomes [26]. All time horizons, as well as both full and partial economic evaluations, were included in the search. However, partial economic evaluations do not include health outcomes; therefore, these are reported separately in the results section. Full economic evaluations include cost-effectiveness analysis (CEA), cost-utility analysis (CUA), and cost–benefit analysis (CBA) [27]. Partial economic evaluations eligible for inclusion comprise cost analysis (CA) and cost consequence analysis (CCA). Outcome measures included in this review are standard in health economic evaluations, primarily the incremental cost-effectiveness ratio (ICER) and incremental cost per quality-adjusted life year (QALY). This review includes studies evaluating costs from all perspectives, including health service, societal, patient, and payer (e.g., insurance-funded healthcare) perspectives. For studies reporting clinical and health economic outcomes separately, only the publications presenting health economic results were included.

## Search strategy and process

The systematic review followed the Joanna Briggs Institute (JBI) guidance for systematic reviews of economic evaluations [28], and was reported in accordance with PRISMA (Preferred Reporting Items for Systematic Reviews and Meta-Analyses) [29]. In addition to providing methods for reviewing evidence from health economic evaluations, JBI also offers a checklist for critical appraisal and standardized steps for synthesis [30]. In this review, the institute’s guidelines were applied consistently throughout the process, with minor adjustments to the data extraction form.

### Information sources

The literature search was performed in the electronic databases PubMed, Scopus, and Health Business Elite. The initial searches were conducted in January 2025 covering the period from 1 January 2015 to 18 January 2025. An updated search was conducted in September 2026 to identify relevant studies published after the initial search. The updated search was performed in the same three databases and covered the period from 1 January 2025 to 16 September 2026.

### Search strategy

The results of the search strategy were uploaded to a reference management system - EndNote (Clarivate). Search terms included MeSH terms and free-text keywords related to digital health, primary care and economic evaluation. The complete database-specific search strategies are provided in Supplementary Appendix 1. An updated search was conducted in September 2026 in the same three databases. The original search terms and Boolean structure were retained. In Scopus, however, the search fields were refined from “All fields” in the original search to “Title, Abstract, and Keywords” in the updated search to increase the specificity of retrieval. No corresponding changes to the search fields were made in PubMed or Health Business Elite.

### Selection process

Duplicates were removed by a reviewer (S.S.) using a reference management system. Following duplicate removal, a preliminary screening was conducted by one reviewer (S.S.) to remove records that were clearly outside the scope of the review (e.g., study protocols, qualitative studies, feasibility studies, paediatric populations, and records unrelated to primary care or economic evaluation). All potentially relevant records proceeded to independent title and abstract screening by two reviewers. Titles and abstracts were then screened by two reviewers working independently (S.S. and L.N.). Full-text copies of relevant studies were retrieved and further assessed for compliance with inclusion and exclusion criteria by two independent reviewers (S.S. and A.J.).

Records identified through the updated search underwent the same study selection process as records identified in the initial search. Following duplicate removal and preliminary title screening by S.S., titles and abstracts were independently screened by two reviewers (S.S. and E.N.), instead of S.S. and L.N. as in the initial search. Full-text assessment followed the same procedures and eligibility criteria as in the initial search. Studies meeting the predefined inclusion criteria were incorporated into the final review.

Classification of primary care was based on organisational context rather than clinical content alone. For example, interventions targeting mild depression delivered in primary care were included, whereas interventions addressing major depression, typically managed within specialist mental health services, were classified as secondary care. Ambiguous cases were reviewed in duplicate and resolved by consensus. There was no disagreement in the selection process, but any potential discrepancies at either stage were planned to be resolved through discussion or reconciliation by a third reviewer (E.N.). Reference lists of included studies were screened manually (one‑step backward citation tracking), where no additional eligible studies were identified.

Search results are displayed in a Preferred Reporting Items for Systematic Reviews and Meta-analyses (PRISMA) flow diagram [29].

### Data collection process

Data from eligible studies was extracted using a slightly modified JBI data extraction form for economic evaluation [28]. The form was piloted on an initial subset of studies, and minor refinements were made following discussion within the study team to ensure clarity and consistency. The modifications involved adding supplementary fields to the extraction sheet since this systematic review focuses on digital health, additional data fields were required to capture the type of intervention and, where available, detailed information on investment costs. Moreover, the data extraction process included documentation of whether threshold values had been applied in the included studies. Details regarding the data extraction form are provided in Appendix 2. The extracted data include both descriptive information about the included studies and results in the form of cost-effectiveness measures. Data extraction was carried out by S.S. and reviewed by A.J.

### Data items

The health outcomes used for data extraction primarily consist of QALYs (quality-adjusted life years), one of the most common measures of health effects in health economics [14]. Since QALY is not a disease-specific measure, it allows comparisons across diagnoses and interventions. QALYs were extracted according to the time horizon reported in each study (typically a lifetime horizon or the study‑defined follow‑up period), and when QALYs were not available, alternative clinical endpoints—such as disease‑specific outcomes, mortality, or other intervention‑relevant health effects—were included.

Differences in costs between the intervention and the comparator are reported in Table 3 in their original currency and reference year. However, to enable meaningful comparisons of cost-effectiveness across interventions, a common currency and price year was applied [31]. The online calculator used was - the CCEMG-EPPI Cost Converter (Campbell and Cochrane Economics Methods Group – Evidence for Policy and Practice Information and Coordinating Centre Cost Converter V 1.7)—which applies purchasing power parity–adjusted exchange rates rather than market exchange rates to enhance cross‑country comparability by accounting for differences in price levels and real resource use [32]. This open access, web-based tool adjusts estimates of cost expressed in one currency and price year to a specified target currency and price year. In this review, Germany was selected as the target country for currency conversion, given its status as a major economy within the European Union. The year 2024 was chosen as the target year, representing the most recent complete year available. For articles originating from countries using a non-domestic currency, currency conversion relied on the exchange rate reported in the respective study.

### Critical appraisal

The quality of the included studies was assessed using the Joanna Briggs Institute’s (JBI) criteria for economic evaluation [30]. The criteria emphasize several key aspects: a clearly defined research question specifying perspective and context; comprehensive description of the intervention and its comparators; identification and measurement of all relevant costs and outcomes; and credible valuation of these components. Time adjustments, such as discounting, must be appropriately applied and justified. Furthermore, incremental cost-effectiveness analysis is required to compare alternatives, and sensitivity analyses should be conducted to address uncertainty. The checklist also considers the relevance of findings for decision-makers and the generalizability of results to other settings. Collectively, these criteria aim to enhance the reliability and comparability of economic evaluations in healthcare research. A complete description of the checklist for critical appraisal, as well as the scores of the included articles, is provided in Appendix 3. An item was rated as “yes”, “no”, “unclear” or “not applicable” based on the information provided in the study. Two reviewers independently assessed the quality of each study and assigned the score (S.S. and A.J.). The results of the quality control were discussed to resolve discrepancies and reach consensus. Any disagreement was resolved by discussion and reconciliation by a third reviewer.

### Effect measures

Effect measures were reported as cost‑effectiveness outcomes, primarily incremental cost‑effectiveness ratios (ICERs), expressed as cost per health outcome gained (e.g. cost per QALY or cost per avoided event), as reported in the included studies.

### Synthesis methods

The synthesis and reporting of results followed the Synthesis Without Meta‑analysis (SWiM) reporting checklist [33]. A narrative analysis of the main characteristics and results of the included studies was conducted, with the synthesis examining findings across different population groups and study designs. Digital health varies widely in design, implementation, cost structures, and outcomes. Narrative analysis allows the integration of findings despite this heterogeneity, whereas statistical pooling would be inappropriate or misleading [34, 35]. Cost‑effectiveness studies often use different economic frameworks (e.g., cost‑utility, cost‑benefit, cost‑consequence analysis). Narrative analysis allows these varied approaches to be compared meaningfully without requiring uniform outcome metrics and help identify recurring themes—for example which types of digital interventions tend to be cost‑effective.

Extracted data were analysed and synthesized to address the review question through the JBI Dominance Ranking Matrix, DRM [36]. This instrument categorizes scientific outcomes by both efficacy and cost, offering a structured approach to summarizing diverse economic evaluation results without requiring complex quantitative pooling. Results are displayed in a 3 × 3 matrix, classifying findings into three categories: strong dominance for intervention, weak dominance for intervention, and non-dominance for intervention. Only full economic evaluations were assessed with the JBI Dominance Ranking Matrix. When QALYs were reported, the direction of the incremental QALY was used to classify health benefit. Alternative health outcomes were used only when QALYs were not available. Thus, a negative health benefit classification reflects a negative incremental QALY, or the corresponding alternative health outcome when QALYs were unavailable, rather than an overall assessment of clinical inferiority. For studies reporting different results across perspectives, all reported outcomes were incorporated into the analysis. Results were presented in summary tables and a dominance ranking matrix, with studies grouped into full and partial economic evaluations.

The protocol specified that meta‑analysis would be explored if sufficiently homogeneous subgroups were identified; however, due to substantial heterogeneity across studies, this approach was deemed inappropriate. The studies differed markedly in economic evaluation type (trial‑based versus model‑based), outcome measures, time horizons, costing perspectives, and analytic frameworks, which precluded the identification of a common effect measure suitable for quantitative pooling. Therefore, a narrative synthesis supported by a dominance matrix was applied to enable a transparent and interpretable synthesis of the evidence. Heterogeneity in reported effects was explored narratively by comparing findings across population groups and study designs. No sensitivity analyses were conducted as part of the synthesis due to substantial heterogeneity across studies; however, reported 95% confidence intervals were extracted for each study, and the conduct of sensitivity analyses was assessed at the study level.

### Reporting bias assessment

Risk of bias due to missing results arising from reporting biases was assessed narratively, in line with Joanna Briggs Institute guidance for systematic reviews of economic evaluations. Formal statistical assessments (e.g. funnel plots) were not applied, as such methods are not considered appropriate for heterogeneous economic evaluations. Assessments were conducted independently by two reviewers (S.S. and A.J.), with disagreements resolved by consensus.

### Certainty assessment

No formal assessment of certainty in the body of evidence was conducted. Instead, the methodological quality of individual studies was appraised using the Joanna Briggs Institute Critical Appraisal Checklist for Economic Evaluations [30], with assessments conducted independently by two reviewers (S.S. and A.J.) and disagreements resolved by consensus.

## Results

### Study selection

The initial systematic search across all databases yielded 4,091 references. In total, 3,551 records were excluded during preliminary screening and 147 duplicates, leaving 393 references for title and abstract screening. The title and abstract screening excluded an additional 277 references. Full-text versions of the remaining 116 publications were obtained and reviewed. Based on these full-text readings, an additional 69 publications were excluded. The main reason for exclusion was non-compliance with the intervention criteria (*n* = 40). A total of 47 articles met the eligibility criteria following the initial search.

An updated literature search identified an additional 658 references: 9 from Health Business Elite, 103 from Scopus, and 546 from PubMed. After removal of 25 duplicates, 633 records remained. Following preliminary title screening, 42 records proceeded to dual independent title and abstract screening, of which 29 were excluded. The remaining 13 publications underwent full-text assessment, resulting in the exclusion of six publications and the inclusion of seven additional articles.

**Fig. 1:**
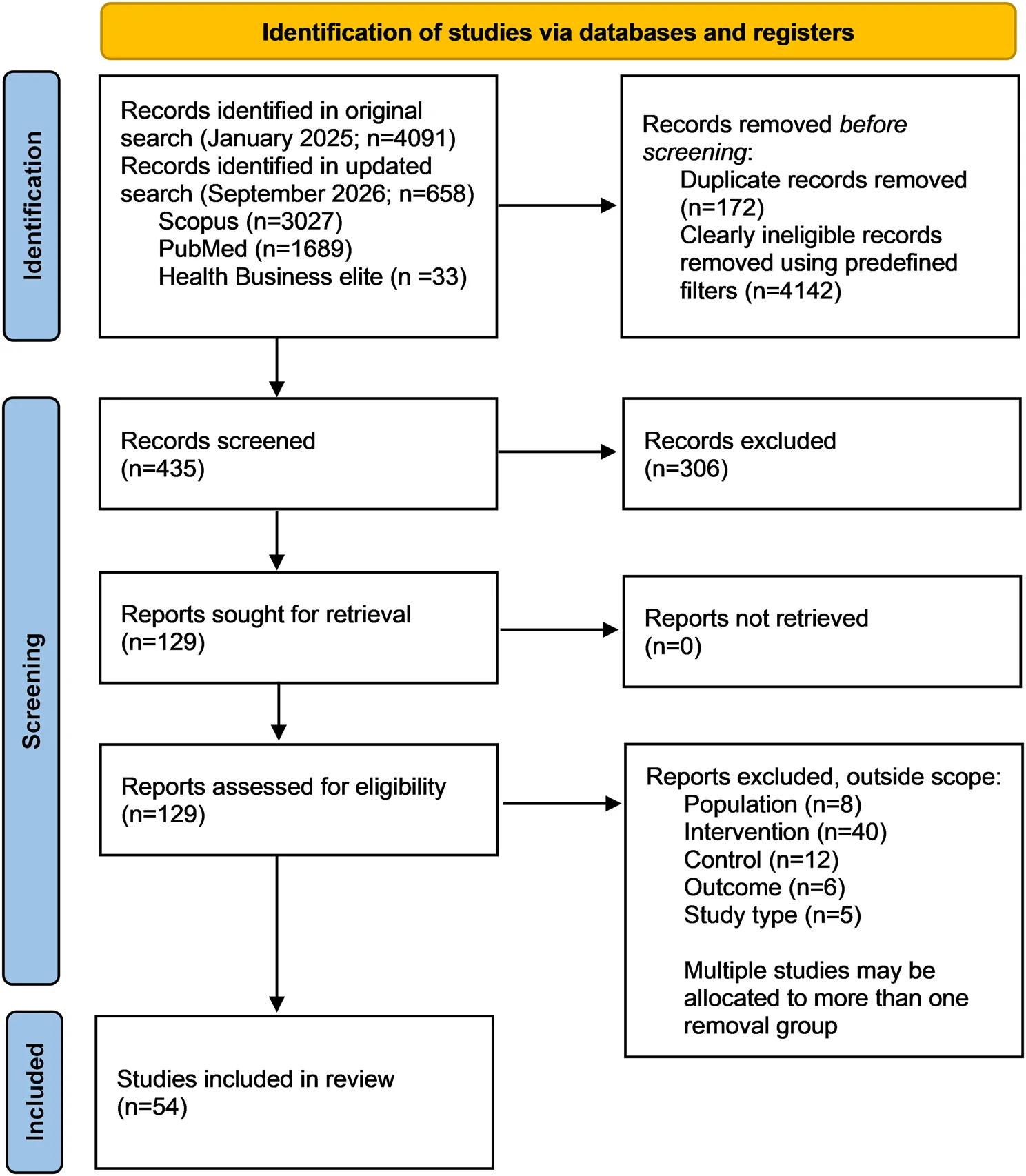
PRISMA 2020 flow diagram illustrating the study selection process for cost-effectiveness of digital health in primary care

Combining the initial and updated searches, a total of 54 articles were included in the final systematic review. The complete reasons for exclusion at full-text assessment are provided in Appendix 4. The study selection process for both the initial and updated searches is presented in the PRISMA 2020 flow diagram [29], shown in Fig. 1.

### Study characteristics

The general characteristics of the included studies are summarized in Table 2. The studies were published from 2015 to 2026 and originated from England (*n* = 10), the Netherlands (*n* = 8), USA (*n* = 8), Australia (*n* = 5), Germany (*n* = 5), Sweden (*n* = 5), Brazil (*n* = 3), Hong Kong (*n* = 2), Belgium (*n* = 1), Denmark (*n* = 1), Finland (*n* = 1), Lebanon (*n* = 1), Nigeria (*n* = 1), Saudi Arabia (*n* = 1), Spain (*n* = 1), and Pakistan (*n* = 1). Of the included studies, the majority were identified as full economic evaluations (*n* = 46), which indicates that both costs and health outcomes were analysed [14]. However, a few studies were partial evaluations, primarily focusing on cost analysis (*n* = 8).

**Table 2:** Main characteristics of included studies evaluating digital health in primary care, including country, target group, type of intervention, occupational group, study type, and characteristics of the economic evaluations (type of analysis, perspective, and time horizon)

Article	Country	Target group of intervention	Type of intervention	Type of digital tool	Occupational group	Study type	Type of economic analysis	Perspective	Time horizon
Partial evaluations									
Chen, 2016 [37]	USA	Overweight and obesity	Intensive Behavioural Counselling	Digital consultations	N/A	Model Based	CA	N/A	10 years
Dahlberg, 2025 [38]	Sweden	Primary care patients in general	Primary care consultations	Digital consultations	Nurses and GP	Retrospective registry-based cost-minimization analysis	CMA	Healthcare	≤1 year
De Queiroz Padilha, 2024 [39]	Brazil	Diabetes and prediabetes	Consultations for diabetes management	Digital consultations	Endocrinologist	Time-Driven Activity-Based Costing	Micro-costing analysis	Healthcare and Patient	2 years
Ekman, 2018 [40]	Sweden	Primary care patients in general	Primary care consultations	Remote consultations	N/A	Cost Analysis	CA	Societal and Payer	N/A
Malik, 2025 [41]	Pakistan	Primary care patients in general	Primary care consultations	Digital consultations	Physicians	Cross-sectional observational survey	CEA	Patient	≤1 year
Sampson, 2022 [42]	England	Depression and anxiety	CBT program	Digital consultations	Team	Before and after quasi-experimental	Interrupted time series analysis	Healthcare	3 years
Winward, 2021 [43]	England	Primary care patients in general	Primary care consultations	Digital consultations	N/A	Retrospective Observational Analysis	CA	Healthcare	2 years
Zaleski, 2025 [44]	USA	Primary care patients in general	Primary care consultations	Digital consultations	Healthcare providers	Retrospective observational episode-based cost analysis	CA	Payer	≤1 year
Full evaluations									
Abi Hana, 2024 [45]	Lebanon	Depression and anxiety	Health intervention to alleviate symptoms of depression, anxiety, and posttraumatic stress	Guided digital health intervention	Nonspecialized staff	Trial-based (RCT)	CEA	Healthcare and Societal	≤1 year
Axelsson, 2020 [46]	Sweden	Depression and anxiety	CBT	Internet-delivered	Therapist	Trial-based (RCT)	CEA and CUA	Societal	≤1 year
Bisson, 2023 [47]	England	Depression and anxiety	CBT-program	Online program	Therapist	Trial-based (RCT)	CEA and CUA	Healthcare	≤1 year
Brouwers, 2021 [48]	The Netherlands	Cardiovascular diseases	Cardiac rehabilitation	Tele-rehabilitation	Team	Trial-based (RCT)	CUA	Societal	≤1 year
Buntrock, 2017 [49]	Germany	Depression and anxiety	Intervention to prevent major depressive disorder in people with subthreshold depression	Web-based guided self-help	Team	Trial-based (RCT)	CEA and CUA	Healthcare and Societal	≤1 year
Chung, 2015 [50]	Hong Kong	Overweight and obesity	Dietary counselling aimed at weight reduction	Telehealth group visits and diet record in a web-based platform	Dietitian	Quasi-experimental design	CEA	N/A	≤1 year
Cruvinel-Júnior, 2024 [51]	Brazil	Diabetes and prediabetes	Therapeutic exercise	Web-based	Physical therapist	Trial-based (RCT)	CEA	Healthcare	≤1 year
Dixon, 2016 [52]	England	Depression and anxiety	Structured depression management programme	Telehealth	Team	Trial-based (RCT)	CEA	Healthcare and Social care	≤1 year
Dixon, 2016 [53]	England	Cardiovascular diseases	Cardiovascular risk‑management programme	Telehealth encounters	Team	Trial-based (RCT)	CEA	Healthcare	≤1 year
Everitt, 2019 [54]	England	Gastrointestinal disease	CBT	Web-based	Therapist	Trial-based (RCT)	CEA	Healthcare and Societal	≤1 year
Faleh Almutairi, 2021 [55]	Saudi Arabia	Diabetes and prediabetes	Diabetes management	Telemedicine care model	Team	Retrospective chart review	CEA	N/A	≤1 year
Fatoye, 2020 [56]	Nigeria	Backpain	Physiotherapy exercise programme	Telerehabilitation	Physio-therapist	Trial-based (RCT)	CUA	Societal	≤1 year
Frederix, 2017 [57]	Belgium	Cardiovascular diseases	Cardiac rehabilitation	Telerehabilitation	N/A	Trial-based (RCT)	CUA	Healthcare and patient	2 years
Freitag, 2024 [58]	Germany	Depression and anxiety	Treatment of depression	Regulated mobile health applications	N/A	Model based	CEA	Payer	5 years
Geraghty, 2024 [59]	England	Backpain	Self‑management programme for low back pain	Internet intervention, with or without support	Physio-therapist	Trial-based (RCT)	CEA	Healthcare	≤1 year
Greving, 2015 [60]	The Netherlands	Cardiovascular diseases	Risk factor management programme	Internet-based	Nurse	Trial-based (RCT)	CEA	Societal	≤1 year
Harvey, 2025 [61]	Sweden	Cardiovascular diseases	Person-centred care	Digital platform	Team	Trial-based (RCT)	CEA and CUA	Societal	2 years
Hermans, 2024 [62]	The Netherlands	Backpain	Patient‑initiated follow‑up model	Asynchronous telemedicine	Rheumatologist	Trial-based (RCT)	CEA	Healthcare and Societal	≤1 year
Kebede, 2025 [63]	Sweden	Depression and anxiety	Person-centred eHealth intervention	Web-based platform	Team	Cross-sectional observational survey	CUA	Societal	≤1 year
Kim, 2024 [64]	USA	Cognitive disorders	Primary care visits	Telehealth visits	N/A	Model based	CEA	Healthcare	10 years
Kloek, 2020 [65]	The Netherlands	Osteoarthritis	Physiotherapy	Blended physiotherapy intervention	Physio-therapist	Trial-based (RCT)	CEA	Healthcare and Societal	≤1 year
Kongstad, 2024 [66]	Denmark	Backpain	Self-management support	App-delivered self-management support	Team	Trial-based (RCT)	CEA	Healthcare and Societal	≤1 year
Koppenaal, 2023 [67]	The Netherlands	Backpain	Physiotherapy	Blended physiotherapy	Physio-therapist	Trial-based (RCT)	CEA	Healthcare and Societal	≤1 year
Kumar, 2018 [68]	USA	Depression and anxiety	CBT	Mobile-based	Therapist	Model based	CEA	Payer and Societal	Lifetime
Lee, 2017 [69]	Australia	Depression and anxiety	CBT	Internet delivered	Team	Model based	CUA	Healthcare	≤1 year
Lee, 2022 [70]	Australia	Depression and anxiety	Stepwise depression management	e-health platform	Nurse	Trial-based (RCT)	CUA	Healthcare and Societal	≤1 year
Lewkowic, 2022 [71]	Germany	Backpain	Therapeutic care	Digital therapeutic care	Physio-therapist	Model based	CUA	Societal	3 years
Little, 2017 [72]	England	Overweight and obesity	Weight management	Web-based	Nurse	Trial-based (RCT)	CEA and CUA	Healthcare	≤1 year
Loohuis, 2022 [73]	The Netherlands	Urinary incontinence	Pelvic floor muscle training programme	App-based treatment	GP	Trial-based (RCT)	CEA and CUA	Societal	≤1 year
Michaud, 2023 [74]	USA	Diabetes and prediabetes	Diabetes prevention program	Digital-based	Health educator	Trial-based (RCT)	CEA	Payer and Societal	≤1 year
Morriss, 2019 [75]	England	Depression and anxiety	CBT	Remote	Therapist	Trial-based (RCT)	CEA and CUA	Healthcare and Societal	≤1 year
Mueller, 2025 [76]	England	Diabetes and prediabetes	Diabetes education + behavioural weight-management	Remote	Dietitian	Trial-based (RCT)	CEA and CUA	Healthcare	≤1 year
Nordyke, 2019 [77]	USA	Diabetes and prediabetes	Behavioural intervention	Digital behavioural intervention	N/A	Model based	CEA	Payer	3 years
Park, 2024 [78]	USA	Diabetes and prediabetes	Diabetes prevention programme	Digital diabetes prevention programme	N/A	Model based	CEA	Healthcare and Societal	10 years
Parker, 2022 [79]	Australia	Overweight and obesity	Preventive health check	Mobile application and telephone coaching	Team	Trial-based (RCT)	CCA	Healthcare	≤1 year
Pelle, 2022 [80]	The Netherlands	Osteoarthritis	Self‑management programme	eHealth application	N/A	Trial-based (RCT)	CEA and CUA	Payer	≤1 year
Priebe, 2024 [81]	Germany	Backpain	Back pain management	App	GP	Trial-based (RCT)	CEA	Payer	≤1 year
Romero-Sanchiz, 2017 [82]	Spain	Depression and anxiety	CBT-based self-help program	Internet-delivered	Psycho-therapist	Trial-based (RCT)	CEA and CUA	Societal	≤1 year
Sjöström, 2015 [83]	Sweden	Urinary incontinence	Pelvic floor muscle training programme	Internet-based program	Uro-therapist	Trial-based (RCT)	CUA	Societal	≤1 year
Smith, 2016 [84]	USA	Diabetes and prediabetes	Diabetes prevention program	Online diabetes prevention program	Team	Model based	CEA	Healthcare and Societal	10 years
Solomon, 2015 [85]	Australia	Depression and anxiety	CBT	Mobile phone- and web program	Psychologist	Model based	CUA	Healthcare	≤1 year
Teixeira, 2024 [86]	Brazil	Smokers	Support for behavioural change	Web-based	Team	Model based	CEA	Healthcare	≤1 year
Toonders, 2023 [87]	The Netherlands	Primary care patients in general	Primary‑care intervention based on cognitive‑behavioural principles	Blended care (both face-to-face and web-based consultations)	Team	Trial-based (RCT)	CEA	Healthcare and Societal	≤1 year
Vomhof, 2026 [88]	Germany	Depression and anxiety	Person-centred eHealth intervention	Primary care + web-based platform	GP and therapist	Trial-based (RCT)	CEA and CUA	Societal	≤1 year
Waller, 2023 [89]	Australia	Diabetes and prediabetes	Self management behaviour change and care support	Text-message intervention	Team	Trial-based (RCT)	CEA and CUA	Payer and patient	≤1 year
You, 2022 [90]	Hong Kong	Depression and anxiety	CBT	Guided internet-based	Psycho-therapist	Model based	CEA	Societal	5 years

Notes: CA; cost analysis, CCA; cost–consequence analysis, CEA; cost-effectiveness analysis, CMA; cost-minimization analysis CUA; cost-utility analysis, CBT; cognitive behavioural therapy, RCT; Randomised Controlled Trial

The majority of studies examined interventions aimed at individuals with depression and anxiety (*n* = 16), followed by patients with diabetes and prediabetes (*n* = 9), interventions targeting back pain (*n* = 7), general primary care patients (*n* = 6), cardiovascular diseases (*n* = 5) and overweight and obesity (*n* = 4), patients with urinary incontinence (*n* = 2), and osteoarthritis (*n* = 2). Intervention target groups represented in only one study comprised smokers (*n* = 1), individuals with cognitive disorders (*n* = 1), and those with gastrointestinal disease (*n* = 1).

Regarding the occupational groups involved in digital interventions across the included studies, a wide range of professions was reported, such as psychotherapists, nurses, physiotherapists, and dietitians. In 41% (*n* = 22) of the studies, physicians were among the participating professional groups, making physicians the most frequently represented profession. In 48% (*n* = 26) of the studies, the intervention was limited to a single occupational group. Furthermore, in several articles (*n* = 9), the occupational group involved in the intervention was not clearly specified.

The predominant study design among the included articles was a trial-based design, randomised controlled trials (RCTs), (*n* = 32). Model-based designs and alternative methodological approaches were equally represented (*n* = 11 each). The alternative approaches included, for example, quasi-experimental designs and retrospective chart reviews.

The costing perspective was primarily from a healthcare perspective in 54% (*n* = 29). This is followed by 50% (*n* = 27) of the articles, which adopt a societal perspective, and subsequently 17% (*n* = 9) that employ a payer perspective. In 7% (*n* = 4) of the articles, a patient perspective is reported. In 35% (*n* = 19) of the articles a multiple perspective was reported and only 6% (*n* = 3) did not report a cost perspective in the economic evaluation. Most studies (*n* = 25) performed a cost-effectiveness analysis (CEA). A considerable proportion of studies (*n* = 12) conducted both CEA and cost-utility analysis (CUA). Only a few studies (*n* = 2) did not employ standard health economic analyses. The time horizon of the included articles was predominantly short-term (*n* = 39), defined as one year or less. In this review, all studies employing a time horizon shorter than 12 months were categorized as “≤1 year,” as cost discounting is not applicable within this timeframe.

### Critical appraisal

The quality of the studies was mainly good and is presented in Table 3. Three of the included studies were structured within a Health Technology Assessment framework, and all three received high quality scores. Common reasons for lower quality score were uncertainties regarding the generalizability of the findings (*n* = 21), inadequate consideration of all issues relevant to users (*n* = 16), and lack of sensitivity analyses (*n* = 12). Since most studies were conducted over a time period of less than one year, discounting was not applicable. These articles are therefore assigned the comment “not applicable” regarding the quality assessment item on discounting. Consequently, the total scores of these articles are reduced, as a lower “yes” response is allocated. A complete list of the critical appraisal based on the JBI checklist is provided in Appendix 3.

**Table 3:** Summary of economic evaluation results from included studies of digital health in primary care, including sample size, cost and effect outcomes (95% confidence intervals), incremental cost-effectiveness ratios (ICERs) converted to 2024 euros (€), authors’ conclusions, and critical appraisal based on the JBI checklist

Article	Sample size	Cost outcome (95% CI), currency and year	Effect outcome (95% CI)	ICER results converted to 2024 Euro (€)	Authors´ Conclusion	Critical Appraisal: JBI
Partial evaluations						
Chen, 2016 [37]	1,121	ROI of CVD Risk Factor Population on Digital IBC: 10 years Sustained weight loss (Completer) 14,660 Partial weight regain (completer) 11,340 Sustained weight loss (ITT) 12,840 Partial weight regain (ITT) 10,250 USD *USD 2015*	Average weight loss (%): Intent-To-Treat 6.9 Completers 7.3	N/A	A digital, remotely delivered IBC program can help seniors at risk for diabetes and cardiovascular disease achieve significant weight loss, reduces risk for diabetes and cardio vascular disease, and achieve meaningful medical cost savings.	5/11
Dahlberg, 2025 [38]	19,697	−50.17 per episode *EUR 2024*	Clinical outcomes assumed equivalent	N/A	Overall, this study supports digital-first models as a cost-effective strategy for managing minor acute conditions in primary care.	
De Queiroz Padilha, 2024 [39]	246	Cost per activity: Teleconsultation 92.41 Face-to-face 88.42 *BRL 2020*	N/A	N/A	The results demonstrate that the incorporation of teleconsultation within the SUS can bring economic advantages depending on the perspective and scenario considered, in addition to being a strategy with the potential to increase access to specialized care in the public network.	6/11
Ekman, 2018 [40]	N/A	Cost: digital consultation 1,960 traditional consultation 3,348 Differences 1,388 *SEK 2017*	N/A	N/A	The digital health care model may be a less costly alternative to the traditional health care model.	5/11
Malik, 2025 [41]	532	−2,800 PKR, year not reported	Incremental difference satisfaction score: +0.9	N/A	Telehealth demonstrates strong acceptance, satisfaction, and economic benefits in Pakistan’s primary healthcare system.	
Sampson, 2022 [42]	10,705	Projected reduction in primary care costs associated with Sleepio rollout per person 4.66(3.20 to 6.13) *GBP year N/A*	N/A	N/A	Sleepio rollout reduced primary care costs. National adoption of Sleepio may reduce primary care costs by £20 million in the first year.	6/11
Winward, 2021 [43]	60,587	Difference in costs per weighted patient: Core services −246 (−34.5) *GBP 2020*	N/A	N/A	Patients with access to 24/7, digital-first primary care incurred significantly lower acute hospital costs.	6/11
Zaleski, 2025 [44]	366,195	10–24% lower costs for 6/11 conditions	N/A	N/A	This real-world study demonstrates the potential of virtual-first care as a lower-cost modality for the treatment of common acute conditions.	
Full evaluations						
Abi Hana, 2024 [45]	1,249	Incremental cost: HC: 28 Soc: −24 *USD 2019*	Incremental effect: Response of depressive symptoms: 0.228 Remission of depressive symptoms: 0.058	Response and remission HC: response = 114 € remission = 446 € Soc: response = dominant, remission = dominant	The findings suggest that SbS is associated with greater health benefits, albeit for higher costs than EUC. seen from the wider societal perspective, there is a substantial likelihood that SbS is not costing more than EUC but is associated with cost-savings as SBS participants become more productive, thus offsetting their health care costs.	8/11
Axelsson, 2020 [46]	204	ΔC: ICB 4,355 FTF-CBT 8,209 *USD 2017*	ΔE: 0.0015 QALY	baseline to post-treatment, a societal gain of 2,6 € million/QALY	In this trial, internet-delivered CBT appeared to be noninferior to face-to-face CBT for health anxiety, while incurring lower net societal costs. The online treatment format has potential to increase access to evidence-based treatment for health anxiety.	8/11
Bisson, 2023 [47]	196	Incremental cost: −572.55 (−1,080.14, −64.96) *GBP 2020*	Incremental effect (QALY): −0.04 (−0.10, 0.01)	N/A	Guided self-help using Spring was not shown to be more cost-effective than face-to-face cognitive-behavioural therapy with a trauma focus although there was no significant difference in accruing quality-adjusted life-years, incremental quality-adjusted life-years −0.04 (95% confidence interval −0.10 to 0.01) and guided self-help using Spring was significantly cheaper to deliver.	11/11
Brouwers, 2021 [48]	300	ΔC: Cardiac telerehabilitation vs Center-based cardiac rehabilitation −3,887 (−4,439) *EUR 2020*	ΔE (QALY): −0.001	N/A	A CTR intervention with relapse prevention was likely to be cost-effective compared with center-based CR.	10/11
Buntrock, 2017 [49]	406	ΔC: HC: 135 (−146 to 418) Soc: 134 (−827 to 1,055) *EUR 2013*	ΔE (QALY): HC: 0.01 (−0.01 to 0.04) Soc: 0.01 (−0.01 to 0.04)	HC: 18,313 €/QALY Soc: 18,177 €/QALY	Our study supports guidelines recommending Web-based treatment for sD and adds that this not only restores health in people with sD, but additionally reduces the risk of developing a MDD. Offering the intervention has an acceptable likelihood of being more cost-effective than enhanced usual care and could therefore reach community members on a wider scale.	10/11
Chung, 2015 [50]	50	Costs/Patient: Intervention 588.39 Control 270.75 *USD 2014*	Observed weight loss (kg): 24 weeks Intervention 10.8 kg. Control 4.7 kg.	254 €/ weight loss (kg)	The face-to-face dietetics model is more cost-effective than the teledietetics model in short-term weight reduction. However, the teledietetics model is more cost-effective in the long-term weight reduction.	8/11
Cruvinel-Júnior, 2024 [51]	62	Incremental cost: 67.16 (−6.22, 140.54) *USD 2022*	Incremental effect (QALY): − 0.07 (−0.11, −0.02)	−1,944 €/QALY	This study demonstrated that SOPeD resulted in significant improvements in foot pain, foot function, and DPN symptoms and severity, although there were no improvements in QALY. The program exhibited comparable costs to usual care and demonstrated cost-effectiveness for foot pain, foot function, and DPN severity and symptoms.	9/11
Dixon, 2016 [52]	609	Incremental mean per-patient cost: 168 (43 to 294) *GBP 2013*	Incremental effect (QALY): 0.001 (−0.023 to 0.026)	20,1376 €/QALY	Incremental costs were high relative to a small incremental QALY gain at 12 months. The intervention is not likely to be cost-effective in its current form.	10/11
Dixon, 2016 [53]	641	Incremental cost: 138 (66 to 211) *GBP 2013*	Incremental effect (QALY): 0.012 (−0.001 to 0.026)	16,487 €/QALY	There is evidence to suggest that the Healthline telehealth intervention was likely to be cost-effective at a threshold of £20 000 per QALY.	10/11
Everitt, 2019 [54]	558	Incremental cost: TCBT: 956 WCBT: 224 *GBP 2015*	Incremental effect (QALY): TCBT compared with TAU 0.0280 QALYs WCBT compared with TAU 0.0172 QALYs	TCBT versus TAU: 33 €/QALY WCBT versus TAU: 11,497 €/QALY TCBT versus WCBT: 78 €/QALY	The complete-case analysis suggests that the therapies are cost-effective from a health-care perspective but this is reduced when using imputation methods. The interventions appear to produce important savings in terms of carer time and, if this is valued, then the interventions do cover their cost, although the societal costs are not significantly statistically different.	10/11
Faleh Almutairi, 2021 [55]	200	Mean difference in cost of treatment: Telemedicine 4,819.76 ± 712.30 Traditional care 4,150.69 ± 910.43 *SAR 2021*	Incremental effect HbA1c: 0.28 (−0.1935 to 0.546)	1,442 €/1% reduction in HbA1c level	Our findings indicated that telemedicine care was cost-effective in the management of type 2 patients with poorly controlled diabetes during the COVID-19 pandemic.	8/11
Fatoye, 2020 [56]	47	Incremental costs: Clinic-based McKenzie therapy N/A Telerehabilitation-based McKenzie therapy −16,000 (−44.26) *USD 2019*	Incremental effect (QALY): Clinic-based McKenzie therapy N/A Telerehabilitation-based McKenzie therapy 0.001 QALYs (0.001 to 0.002)	ICER Clinic-based McKenzie therapy N/A Telerehabilitation-based McKenzie therapy Dominant	The findings of the study suggested that telerehabilitation for people with NCLBP was cost saving.	5/11
Frederix, 2017 [57]	126	Total average cost per patient: Intervention 3,262 Control 4,140 Incremental cost −878 *EUR 2015*	Incremental effect (QALY): 0.22	− 4,943 €/QALY	A combined telerehabilitation and centre-based programme, followed by transitional telerehabilitation induced persistent health benefits and remained cost-efficient up to 2 years after the end of the intervention. A partial decline of the benefits originally achieved did occur once the tele-intervention ended.	8/11
Freitag, 2024 [58]	4,977 000	Treatment 1: Without DiGA scenario 7,933 Treatment 2: With DiGA scenario standard of care 7,980 Treatment 3: With DiGA future scenario 9,469 *EUR 2023*	Effect (QALY): Treatment 1: Without DiGA scenario 2.16478 Treatment 2: With DiGA scenario standard of care 2.16538 Treatment 3: With DiGA future scenario 2.18367	Treatment 2: 83,139 €/QALY Treatment 3: 85,094 €/QALY (comparator Treatment 1)	Based on our CMM, we concluded that DiGA for the depression on the one hand can help to improve the quality of life of people suffering from depression, but on the other hand are associated with noticeable extra costs and hence currently represent no dominant health strategy.	10/11
Geraghty, 2024 [59]	825	Incremental cost: SupportBack −16 (−128 to 95) SupportBack and telephone support 96 (−14 to 206) *GBP 2021*	Incremental effect (QALY): SupportBack: 0,011 (−0,0014 to 0,039) SupportBAck and telephone support: 0,013 (−0,011 to 0,037)	SupportBack Dominates SupportBack and telephone support: 71,110 €/QALY	The SupportBack internet interventions did not significantly reduce low back pain-related disability over 12 months compared with usual care. They were likely to be cost-effective and safe.	10/11
Greving, 2015 [60]	330	Cumulative mean costs: Intervention 4,859 Control 5,078 Differences −219 (−2,301 to 1,825) *EUR 2009*	Adjusted incremental effect (QALY): −0.014 (−0.017 to 0.041)	N/A the intervention had a probability of 65% to be cost-effective compared with usual care (at a €20,000/QALY threshold value)	An internet-based, nurse-led intervention in addition to usual care to improve vascular risk factors in patients with a clinical manifestation of a vascular disease does not result in a QALY gain at 1 year, but has a small effect on vascular risk factors and is associated with lower costs.	10/11
Harvey, 2025 [61]	222	Incremental costs: − 68,533.12 (−86,827.63 to 50,238.6) *SEK 2021*	Incremental effect (QALY): +0.0469 (0.0252 to 0.0686)	Dominant; lower costs and higher QALYs	The results of this CEA demonstrated that a remote PCC add- on intervention for people with COPD and/or CHF had lower healthcare costs and higher health related quality of life compared with usual care.	
Hermans, 2024 [62]	200	Incremental costs: adjusted HC: −180 Soc: 575 *EUR 2023*	Incremental effect (QALY): HC: 0.004 Soc: 0.004	HC: dominated by usual care Soc: 135,808 €/QALY	Patient-initiated follow-up and asynchronous telemedicine resulted in significant and clinically meaningful reductions in rheumatology visits. This is not at the expense of health outcomes and saved health-care costs.	10/11
Kebede, 2025 [63]	206	Incremental costs: −20,296 per patient *SEK 2019*	Incremental effect (QALY): +0.0054	−411,580 €/QALY	Add-on person-centered eHealth interventions improves upon the usual care only for patients on sick leave for CMDs. This approach has proven to be cost-effective in managing CMDs and supporting better outcomes for patients on sick leave.	
Kim, 2024 [64]	91	Incremental cost, mean: In-person visit 503 Telehealth visit 436 *USD 2022*	Modified ZBI-12 (Total 0–100): Mean In-person visit 60.58 Telehealth visit 60.37	− 267 €/ modified ZBI-12	Among urban-residing non-Hispanic White individuals, the ICER per modified ZBI-12 indicates a USD 9.44 cost-saving with telehealth visits; among urban-residing racial minorities, the ICER per modified ZBI-12 indicates a USD 29.26 cost-saving with in-person visits; among rural residents, the ICER per modified ZBI-12 indicates a USD 320.93 cost-saving with telehealth visits.	7/11
Kloek, 2020 [65]	208	Adjusted mean cost difference: HC: − 792 (−2,720 to 1100) Total: − 529 (−3,315 to 2057) *EUR 2015*	ΔE (QALY): 0.01 QALYs (− 0.03 to 0.04)	HC: −101,696 €/QALY Total: −67,926 €/QALY	E-Exercise itself was significantly cheaper compared to usual physiotherapy in patients with hip and/ or knee osteoarthritis, but not cost-effective from the societal- as well as healthcare perspective. The decision between both interventions can be based on the preferences of the patient and the physiotherapist.	10/11
Kongstad, 2024 [66]	297	Incremental cost: HC: 230 (−136 to 595) Soc: 589 (−2,120 to 3,298) *EUR 2022*	Incremental effect (QALY): 0.03 (0.01 to 0.05)	HC: 6,649 €/QALY Soc: 17,058 €/QALY	From a healthcare perspective, the selfBACK interventions likely to represent a cost-effective treatment for people with LBP.	10/11
Koppenaal, 2023 [67]	208	Incremental cost: Soc: 994 (−1,002 to 3,320) HC:47 (−81 to 192) *EUR 2020*	Incremental effect (QALY): Soc: 0.02 (−0.02 to 0.06) HC: 0.02 (−0.02 to 0.06)	Soc: 57,683 €/QALY HC: 2,627 €/QALY	The stratified blended physiotherapy intervention e-Exercise LBP is neither more effective for improving physical functioning nor more cost-effective from societal or health care perspectives compared with face-to-face physiotherapy for patients with nonspecific LBP.	10/11
Kumar, 20,218 [68]	100,000	Incremental cost. Reduction in Costs, million $ Soc: 2,234 Payer: 339 *USD 2015*	Incremental effect (QALY): Soc: 34,108 Payer: 34,108	N/A	Mobile CBT may lead to improved health outcomes at lower costs than traditional CBT or no intervention and may be effective as either prevention or treatment.	10/11
Lee, 2017 [69]	N/A	Total costs, base case excl. hospitalization Mindspot 472.75 Routine care 488.04 *AUD 2014*	QALY: Mindspot 0,816 Routine care 0,798	Mindspot was found to be dominant	The iCBT treatments provided by MindSport were highly cost-effective in comparison with usual care in Australian setting.	10/11
Lee, 2022 [70]	1,868	Ratio of mean costs between arms at 12 months 0.98 (0.88 to 1.08) *AUD 2019*	Incremental effect (QALY): 0.011 (0.001 to 0.022)	HC: Dominant Soc: Dominant	Target-D is likely to represent good value for money for health care decision makers.	10/11
Lewkowicz, 2022 [71]	10,000	The digital therapeutic care app strategy cost 121.59 more per patient *EUR 2021*	Incremental effect (QALY): 0.0221	6,378 €/QALY	The current reimbursement rate for a digital therapeutic care app in the statutory health insurance can be considered a cost-effective measure compared to TAU.	11/11
Little, 2017 [72]	818	Control 424 (338 to 515) POWeR+F 447 (358 to 544) POWeR+R 388 (315 to 468) Incremental cost: POWeR+F 23 (−105 to 152) Incremental cost POWeR+R −36 (−154 to 81) *GBP 2014*	Mean QALY 12 months: Control 0.78 (0.74 to 0.82) POWeR+F 0.83 (0.80 to 0.86) POWeR+R 0.82 (0.78 to 0.86)	POWeR+F vs. control 27 €/ kg of weight lost POWeR+R vs. control −37 €/ kg of weight lost	A web-based behavioural programme and brief support results in greater mean weight loss and 10% more participants maintain valuable weight loss; it achieves greater enablement and fewer participants undertaking other weight-loss activities; and it is likely to be cost-effective.	10/11
Loohuis, 2022 [73]	262	Incremental cost: −161 (−180 to −151) *EUR 2017*	Incremental effect (QALY): −0.025	8,052 €/QALY	App-based treatment is a cost-effective alternative to care-as- usual for women with UI in Dutch primary care.	10/11
Michaud, 2023 [74]	599	Incremental cost: Private payer: 379 Soc: −1,638 *USD 2020*	Incremental effect HbA1c (%): Private payer −0.08 Soc: −0.08	Private payer 6,423 €/ HbA1c% Soc: cost-saving	The d-DPP was cost-effective and offers the prospect of high scalability and sustainability due to its program features and delivery modes, which can be easily translated to other settings.	10/11
Morriss, 2019 [75]	156	Δ C: −1,064 (−2973 to 845) *GBP 2017*	Δ E (QALY): At 12 months: 0.07 (0.07 to 0.21)	−21,818 €/QALY	RCBT may be an easy-to-implement intervention to improve clinical outcome and save costs in one group of repeat users of unscheduled care.	9/11
Mueller, 2025 [76]	577	Incremental cost: −232 *GBP 2022*	Incremental effect (QALY): −0.001 (−0.02 to 0.03)	292,354 €/QALY	A commercial behavioural weight management programme combined with remote dietary counselling after diagnosis of type 2 diabetes did not improve HbA1c up to 12 months post intervention in this trial. The intervention could help people achieve weight loss and be cost-effective compared with current standard National Health Service care.	
Nordyke, 2019 [77]	N/A	Savings in T2DM are estimated at $83 per participant per month (PPPM) in year 1 and rise to 174 to 178 in years 2 and 3, respectively. Year 1 savings in HTN are estimated at 70 PPPM rising to 113 and 107 in years 2 and 3, respectively. *USD 2018*	N/A	N/A	The DTxs studied may provide substantial cost savings, in part by reducing the use of conventional medications. Clinical inertia may limit the full cost savings of DTxs.	10/11
PARK, 2024 [78]	N/A	Incremental cost: Soc: −3,672 HC: −2,990 *USD year N/A*	Incremental effect (QALY): 0.08	d-DPP dominated in-person DPP	The d-DPP is a cost-effective alternative to in-person lifestyle interventions for preventing the development of T2D among individuals with prediabetes in the United States.	10/11
PARKER, 2022 [79]	215	Mean diff GP, specialist & PBS costs. excluding outlier Control 89 (−257, 435) Intervention 148 (−205, 502) *AUD 2020*	Quality of life change: Control 0.87 (0.16) Intervention 0.90 (0.11)	N/A	While the intervention was associated with some improvements in health literacy and diet, substantial differences in other outcomes were not observed.	4/11
PELLE, 2022 [80]	427	Total health care costs during follow-up Intervention 462 (80) Control 503 (79) Difference −22 (−36, −3) *EUR 2018*	Incremental effect (QALY): 0.00 (−0.00, 0.01)	N/A	This economic evaluation showed that costs were lower for the dr. Bart app group compared to the group who received usual care.	9/11
Priebe, 2024 [81]	1,237	Total costs of the Rise-uP group decreased from 411.65 to 372.45 per patient within twelve months, while the costs of CG increased by 207.75 *EUR 2020*	N/A	Cost savings of €590.58 per point reduction on the NRS pain scale in favour of the Rise-uP group	The Rise-uP approach with a medical multimodal back pain app as the central element of digital treatment demonstrates both, clinical and economic superiority compared to standard of care in the management of NLBP.	8/11
Romero-Sanchiz, 2017 [82]	296	Incremental cost: tau vs totally self-guided Internet-based program −644.11 low-intensity therapist-guided Internet-based program vs tau −479.20 *EUR 2014*	Incremental effect (QALY): tau vs totally self-guided Internet-based program 0.0567 low-intensity therapist-guided Internet-based program vs tau 0.0751	tau vs totally self-guided −258 €/QALY Internet-based program low-intensity therapist-guided Internet-based program vs tau −159 €/QALY	The results of this study indicate that both Internet-based CBT interventions showed cost utility and cost effectiveness compared with a control group.	9/11
Sjöström, 2015 [83]	250	Incremental cost: Internet-based vs. postal 0.3 Internet-based vs. no treatment 322 *EUR 2010*	Incremental effect (QALY): Internet-based vs. postal 0.0014 Internet-based vs. no treatment 0.0104	Internet-based vs. postal 244€/QALY Internet-based vs. no treatment38 €/QALY	An Internet-based treatment for SUI is a new, cost-effective treatment alternative to usual care.	9/11
SMITH, 2016 [84]	50	Incremental cost: HC: 591 SOC: 1,209 *USD 2010*	Incremental effect (QALY): HC: 0.0412 SOC: 0.0412	HC: 15,782 €/QALY SOC: 32,255 €/QALY	The ODPP may offer an economical approach to combating overweight and obesity.	10/11
Solomon, 2015 [85]	N/A	Incremental cost: myCompass versus TAU 158 myCompass versus CBT 1,996 *AUD 2014*	Incremental effect (QALY): myCompass vs TAU: −0.02 myCompass vs CBT: 0.03	myCompass vs TAU: −5,880 €/QALY myCompass vs CBT: 2,070 €/QALY	Internet-based interventions can provide cost-effective access to treatment when provided as part of a stepped care model. Widespread dissemination of Internet-based programs can potentially reduce demands on primary and tertiary services and reduce unmet need.	10/11
Teixeira, 2024 [86]	5,400	Incremental cost: service provider: −17,743.06 Unified Health System: −16,250.06 *BRL 2020*	Incremental effect: (Engagement in cessation service) provider: 0.05 Unified Health System: 0.05	Service provider: −58,397 €/ Engagement in cessation Unified Health System: −14,5068 €/ Engagement in cessation	The decision-making models of the cost-effectiveness analysis showed that in treating smoking, using electronic tools results in cost savings. In addition to being cheaper, the Pare de Fumar Conosco software provides the best health result compared to the standard of care.	10/11
Toonders, 2023 [87]	160	Δ C: HC: 848 (443 to 1,612) Soc: −333 (−1816 to 1,066) *EUR 2020*	ΔE (QALY): HC: − 0.01 (− 0.07 to 0.05) Soc: − 0.01 (− 0.07 to 0.05)	HC: − 101,124 €/QALY Soc: 39,660 €/QALY	Blended primary care intervention was not found to be cost-effective compared to usual care.	10/11
Vomhof, 2026 [88]	376	Incremental cost: −866 (−3,523 to 1,911) *EUR 2021*	Incremental effect (QALY): 0.015 (−0.010 to 0.041)	Dominant	While the PROVIDE-C intervention is less likely cost-effective 6 months after baseline, it was likely cost-effective 12 months after baseline.	
Waller, 2023 [89]	395	Incremental cost: 44 (−214, 325) *AUD 2018*	Incremental effect (QALY): −0.004 (−0.02, 0.01)	an ICER could not be calculated as mean QALYs were higher in the control group than in the intervention group	DTEXT was low cost and potentially scalable, but only had a low to moderate probability of being effective and cost saving.	9/11
You, 2022 [90]	N/A	Increment cost: − 145 *USD 2022*	Incremental effect (QALY): 0.0039	N/A	Guided iCBT appears to be cost-saving and effective for management of university students with mild symptoms of anxiety from the societal perspective of Hong Kong.	10/11

Notes: ΔC; Delta costs, ΔE; Delta effect, AUD; Australian dollar, BRL; Brazilian real, EUR; Euro, HC; Health Care, GBP; Great British Pound, ITT; Intention-to-Treat, JBI; Joanna Briggs Institute, PKR; Pakistani rupees, ROI; Return of investment, SAR; Saudi Riyal, Soc; societal, SEK; Swedish Krona, TAU; treatment as usual, USD; US-dollar

### Results of individual studies

### Results of syntheses

#### Primary outcome measurements

Of the 46 full economic evaluations included in the review, 36 (78%) reported health outcomes in terms of QALYs. In contrast, studies that do not utilize QALY report alternative endpoints, such as weight reduction, changes in HbA1c, improvement in depressive symptoms, and scores on the Modified ZBI-12.

#### Target groups of intervention

Digital models for weight management [68], diabetes prevention [70, 73], and cardiovascular risk reduction [54, 61] show clinical benefits and offer potential for long-term medical cost savings. Similarly, teleconsultation and telerehabilitation strategies provide economic benefits alongside sustained health outcomes [54, 57, 59, 61, 75, 76]. Across the reviewed literature, a consistent pattern emerges suggesting that digital and Internet‑based psychological interventions offer meaningful clinical benefits, though their cost‑effectiveness varies depending on intervention type, target population, and implementation context. Several studies examining Internet‑delivered cognitive behavioural therapy (iCBT) indicate that such formats can maintain clinical effectiveness while reducing overall societal costs [44, 64, 65, 71, 77, 84]. Mental health interventions delivered online, including iCBT and guided self-help, are typically non‑inferior to face‑to‑face therapy and may reduce societal costs by mitigating productivity losses and improving accessibility to evidence‑based care. Digital mental health interventions can be cost‑effective, particularly when integrated within stepped‑care models. Heterogeneity in study findings was examined narratively and was largely explained by differences in target population, analytical perspective, and implementation context.

#### Included costs

The included studies show that costs for digital health can be divided into initial investment costs and recurrent operating costs. Investment costs include the development of digital platforms, apps, and websites, as well as the purchase of hardware such as smartphones, tablets, and servers. These costs are often high but one-time in nature and are sometimes excluded from economic analyses, particularly when development has been externally funded or occurred prior to the study. Licenses and initial purchases, such as materials and medical equipment, also appear in several analyses. Research and development costs are often excluded as they are not considered relevant for operational purposes.

Operating costs are required to run and sustain the intervention and include hosting, maintenance, and upgrades of platforms, administration and technical support, as well as personnel resources for training, supervision, and patient contact. Communication costs (telephone, SMS, internet data) and overhead costs for facilities and equipment are also mentioned. Cost calculations vary depending on perspective: some studies include indirect costs such as productivity losses and travel, while others limit the analysis to direct healthcare and technology costs. Scalability is an important factor; fixed IT costs increase with scale-up, but the marginal cost per user decreases, creating economies of scale. Several studies use market prices or license fees as a basis, while others perform detailed resource-based calculations.

A 95% confidence interval (95% CI) is presented for cost outcomes and effect outcomes in Table 3, provided that these are clearly reported in the respective study. N/A is applied where a calculated ICER was not reported in the article, even though both costs and effects were presented.

The majority of studies (*n* = 38) included a threshold value, typically a willingness-to-pay (WTP), in the cost-effectiveness analysis. A calculation of net monetary benefit is presented in 20% (*n* = 11) of the articles.

#### Classification of studies using the dominance ranking framework

The dominance ranking framework classifies interventions based on their relative costs and outcomes, indicating whether the evidence favours adoption of the intervention, suggests unclear or mixed cost‑effectiveness, or supports rejection of the intervention [36]. In accordance with the predefined outcome hierarchy, QALYs were used to determine the direction of health benefit when reported, while other measures of health benefit were used when QALYs were not available. Of the 49 dominance ranking results, 39 were classified using QALYs and 10 using other health outcome measures because QALY estimates were not reported. The framework and the dominance ranking results for the included studies are presented in Table 4.

**Table 4:** JBI dominance ranking matrix of all included full economic evaluations for digital health (*n* = 49)

Cost	Health benefit	Implication for decision makers	No. of studies
+	-	Reject intervention	3* [51, 87, 89]
0	-	Reject intervention	
+	0	Reject intervention	
-	-	Unclear	7** [47, 48, 60, 64, 73, 76, 87]
0	0	Unclear	
+	+	Unclear	17*** [45, 49, 50, 52–55, 58, 62, 66, 67, 71, 74, 79, 83–85]
-	0	Favour intervention	1 [80]
0	+	Favour intervention	
-	+	Favour intervention	21**** [45, 46, 56, 57, 59, 61–63, 65, 68–70, 72, 74, 75, 78, 81, 82, 86, 88, 90]

(+) The intervention is costlier or more beneficial than the comparator; (0) The intervention has a cost or outcome/benefit equal to the comparator; (-) The intervention is less costly or less beneficial than the comparator

*One of the studies is also classified under other categories from an alternative perspective

**One of the studies is also classified under other categories from an alternative perspective

***Three of the studies is also classified under other categories from an alternative perspective

****Three of the studies is also classified under other categories from an alternative perspective

In this review, 22 studies were classified as “favour intervention”, 24 as “unclear” and three “reject intervention”. Among the studies categorized as ‘favour intervention’, 16 out of 22 articles were RCTs, one was a cross-sectional observational survey, while the remainder were model-based studies. Nearly half of the studies with this result focused on interventions targeting depression and anxiety (10 out of 22). Three studies reported alternative classifications depending on the analytical perspective. Of the 22 studies favouring the intervention, 21 reported both lower costs and greater health benefits and were therefore classified as dominant. Ten of these 21 studies targeted depression and anxiety. One additional study reported lower costs with equivalent health benefits.

Among the 24 studies classified as “unclear”, 17 reported greater health benefits at higher costs, while seven reported lower costs accompanied by lower health benefits. For studies reporting QALYs, these classifications reflect the direction of incremental QALYs rather than an overall assessment of clinical effectiveness. The cost-effectiveness of interventions in this category therefore depends on the value assigned to the incremental gains or losses in health relative to the corresponding differences in costs.

The three articles reporting results in which the digital health incurs a higher cost and simultaneously demonstrates poorer effectiveness compared to the control were all RCTs, and two of these focused on interventions for patients with diabetes [51, 89]. The third examined general primary care patients [87]. The article by Toonders et al. [87] also reports an alternative finding, indicating that the intervention results in lower costs when analysed from a societal perspective.

### Reporting biases

Risk of bias due to missing results was assessed at the synthesis level using the Joanna Briggs Institute critical appraisal checklists [30]. Full assessment results are provided in Appendix 3; no funnel plots or statistical tests for reporting bias were conducted, and overall risk was judged to be low to moderate.

### Certainty of evidence

Certainty of evidence was informed by study quality as assessed using the Joanna Briggs Institute critical appraisal checklists [30]; no separate certainty framework was applied. Full results are presented in Appendix 3.

## Discussion

### Principal findings

This review provides a comprehensive overview of economic evaluations of digital health within primary care and first-line services. The reviewed studies, published between 2015 and 2026 across diverse countries, predominantly used trial-based designs and full economic evaluations, mainly from a healthcare perspective. Most interventions targeted mental health conditions such as depression and anxiety, followed by diabetes and musculoskeletal disorders, and involved a range of professional groups, with physicians represented in more than one-third of the studies. The main findings of this review suggest that digital health generally demonstrates potential in terms of cost-effectiveness, particularly in mental health contexts, where most analyses report improved outcomes and reduced costs.

### Cost-effectiveness

The accumulated evidence suggests that digital and remotely delivered health in primary care is associated with favourable economic outcomes across a broad range of clinical areas, with the majority of studies reporting cost-effectiveness alongside improved or maintained health outcomes. These findings are consistent with previous research [18, 91, 92]. While some programs yield modest improvements in quality-adjusted life years (QALYs), they remain cost-efficient owing to lower delivery costs and scalability. Moreover, ongoing debates concern the suitability of QALYs as an outcome measure in evaluations of digital health, as previous research indicates that this metric may require complementary measures capturing both health‑related and non‑health‑related effects [93]. The findings from the updated search were consistent with and reinforced the overall pattern observed in the review, with all four additional full economic evaluations favouring the digital health intervention and the three partial economic evaluations reporting lower costs compared with usual care.

However, digital health is not always superior to traditional care in every context; cost-effectiveness often depends on the perspective (healthcare vs. societal), time horizon, and implementation model [94, 95]. During the critical appraisal, it was frequently noted that the studies offered a comprehensive account of methodological choices as well as relevant costs and effects associated with the interventions. Nevertheless, variations in the approaches adopted across studies were apparent. A key challenge in the literature concerns the heterogeneity in methods for incorporating intervention costs, which affects comparability and conclusions about cost-effectiveness [96] which has also been noted in previous research [92]. Some analyses exclude development and research costs, while others include them proportionally per user or calculate based on market prices. Additionally, perspectives differ—some studies restrict analyses to direct healthcare costs, while others include indirect costs such as productivity losses and travel. These methodological inconsistencies complicate the ability to draw generalizable conclusions and may introduce bias into economic evaluations [97].

In the reviewed articles, initial investment costs were substantial but typically one-time, whereas operating costs were recurring and shaped by technical maintenance, personnel input, and organizational resources. Variations in cost calculations underscore the need for clearly defined boundaries and perspectives in economic evaluations of digital health [98, 99]. CHEERS-AI (Consolidated Health Economic Evaluation Reporting Standards for Interventions That Use Artificial Intelligence) offers structured guidance for reporting economic evaluations, encompassing both digital and AI-based interventions [100]. The standard mandates transparent reporting of all cost-related parameters, including explicit definitions of cost categories and specification of included components, such as development, implementation, and maintenance. It also calls for explicit clarification of the chosen perspective (e.g., societal, healthcare system) and the time horizon applied in the analysis. To ensure methodological rigor, cost calculations should be standardized to enable comparability across studies [101]. Nevertheless, a wide range of approaches persists in conducting such studies. Prior reviews of the literature have also acknowledged this challenge [18, 102]. This is particularly relevant for digital health, where initial investments and recurring operational costs vary considerably across studies [103]. There are additional methodological challenges associated with digital health, as the rapid pace of technological evolution complicates the application of traditional study designs [103]. Furthermore, digital health often presents a broader benefit profile than conventional care, adding complexity to cost and outcome assessments [93, 98].

The choice of perspective in economic evaluations—whether healthcare system, payer, societal, or patient—significantly influences the selection of costs and benefits [94]. In the context of digital health, this is further complicated by the involvement of multiple stakeholders beyond traditional healthcare providers, such as technology developers, insurers, employers, and patients themselves [98]. Valuing digital health necessitates recognition of this multi-stakeholder environment, where each actor may prioritize different dimensions of value [104]. It is therefore encouraging to observe that the majority of studies have adopted multiple perspectives when conducting their analyses.

## Strengths and limitations

A major strength of this systematic review lies in the convergence of findings across multiple studies, representing a substantial contribution to the evidence base. Consistency among independent analyses minimizes the risk that observed effects result from random variation, methodological bias, or context-specific factors. Such alignment enhances both internal validity and external generalizability, indicating that the intervention’s effectiveness remains robust across diverse settings and populations. Several studies offer evidence of strong dominance in favour of the intervention. The group categorized as “Unclear” indicates that cost-effectiveness interpretation is strongly contingent upon the accepted willingness-to-pay threshold. In these cases, the intervention may demonstrate superior health outcomes but at a higher cost, or alternatively, reduced effectiveness accompanied by lower costs. Consequently, whether the intervention is considered cost-effective depends on the willingness-to-pay threshold for gaining additional health benefits per unit of cost [98]. This uncertainty reflects the trade-off between greater clinical benefit and increased expenditure, highlighting the importance of context-specific thresholds in economic evaluations. A well-documented sensitivity analysis was reported in 42 of the 54 included articles, thereby strengthening the reliability of the reported results [31].

There are certain limitations in this systematic review. One challenge within the field of digital health is the use of relevant search terms, as the area is evolving rapidly and the terminology changes quickly [105]. For example, the term telemedicine is common among studies in digital health [106] and is therefore a search term included in the database searches. A variety of keywords were employed to achieve as broad a search result as possible. Since the broad search strategy generated an extensive number of hits, grey literature was deemed unnecessary to include. However, this decision is open to debate, as additional relevant findings for a systematic review could potentially be identified in grey literature sources [31].

This review excluded home-monitoring tools, despite their increasing relevance within primary care. The scope was limited to digital interventions formally integrated into primary care service delivery, which may underestimate the broader role of digital health in supporting patient-led care and system-level efficiencies. The restriction of the search to English and Nordic languages may have excluded relevant evidence from other regions, although studies from several non–English-speaking countries were nevertheless included, indicating that evidence from diverse health system contexts is to some extent represented.

Including both model-based and trial-based economic evaluations in a systematic review entails methodological challenges. These two approaches differ fundamentally in design: trial-based studies rely on observed data from clinical trials, often with short time horizons, while model-based evaluations extrapolate outcomes over longer periods using assumptions and secondary data [26, 107]. This results in heterogeneity in cost estimates, perspectives, and analytic frameworks, rendering quantitative synthesis (e.g., meta-analysis) inappropriate and increasing the risk of biased conclusions. Nevertheless, there are clear advantages to incorporating both types of studies [25]. Trial-based evaluations provide high internal validity and real-world resource use data, while model-based studies offer long-term projections and the ability to capture outcomes beyond the trial period. Integrating these approaches in a narrative synthesis can enhance understanding by illustrating how short-term empirical findings align—or diverge—from modelled long-term projections. This comprehensive view supports more informed decision-making [96], especially for interventions like digital health solutions where initial costs and scalability effects unfold over time. Moreover, employing the dominance matrix enables a visual summary of diverse economic analyses with varying outcome measures, which would otherwise not be feasible within a quantitative meta-analysis framework [108]. The studies included in the dominance ranking matrix used a variety of health outcome measures. To maximise comparability, QALYs were prioritised whenever available. For studies not reporting QALYs, the primary effectiveness outcome reported by the study authors was used. Consequently, the classifications should be interpreted as a structured summary of the direction of costs and effects rather than as a direct quantitative comparison across studies.

This systematic review covers a diverse range of target populations and age groups. However, there is a potential risk that the individuals included in the studies were selected based on criteria that facilitated the use of digital health, which may result in an underrepresentation of patients with lower levels of digital literacy. A previous systematic review focusing on older adults found limited evidence of cost-effectiveness for Mobile Health (mHealth) [109]. Including diverse patient groups in a systematic review on the cost-effectiveness of digital health can be challenging for several reasons. First, patient populations may vary considerably in demographic characteristics, health conditions, and care needs, introducing heterogeneity in both clinical and cost outcomes. Second, the included studies employed different economic evaluation methods, complicating comparisons across groups. These factors complicate the synthesis and may limit the generalizability of findings across different populations and types of interventions [35]. Nevertheless, conducting a broad review provides important advantages. By synthesizing existing evidence across primary care, this review offers a comprehensive overview of current research. It also enables decision-makers to grasp the range of cost-effectiveness and outcomes reported for different patient groups [96], thereby supporting more inclusive and evidence-based strategies for implementing digital health in primary care settings.

This review is innovative in its focus on the cost‑effectiveness of digital health in primary care. Unlike previous reviews, it systematically synthesises trial‑ and model‑based economic evaluations. The review advances the field by clarifying the economic evidence base and has practical implications for decision‑makers considering the implementation of digital health in primary care.

## Conclusion

The findings provide substantial evidence that digital health in primary care can be cost-effective across a broad range of clinical areas. Although the included studies varied considerably in methodological approaches, economic perspectives, time horizons, and outcome measures, the majority reported favourable economic outcomes. The consistency of this pattern across diverse study designs and settings strengthens the evidence supporting digital health as an economically viable alternative or complement to conventional care. Nevertheless, methodological heterogeneity limits direct comparability, and uncertainty remains in less extensively studied clinical areas. These findings support the consideration of digital health in healthcare decision-making while emphasising the need for context-specific assessment of costs, outcomes, and implementation conditions.

## Electronic supplementary material

Below is the link to the electronic supplementary material.

Supplementary Material 1

Supplementary Material 2

Supplementary Material 3

Supplementary Material 4

## Data Availability

All data extracted and analysed during this study are included in this published article and its supplementary information files.

## Abbreviations

CA: Cost Analysis
CCA: Cost-Consequence Analysis
CBA: Cost-Benefit Analysis
CEA: Cost-Effectiveness Analysis
CUA: Cost-Utility Analysis
DID: Difference-in-Differences
ICER: Incremental Cost-Effectiveness Ratio
JBI: Joanna Briggs Institute
NMB: Net Monetary Benefit
PICO: Population–Intervention–Comparison–Outcome
QALY: Quality-Adjusted Life Year
PRISMA: Preferred Reporting Items for Systematic Reviews and Meta-Analyses
WTP: Willingness-To-Pay
