# Supplementary material for: Cost-effectiveness of digital health in primary care: a systematic review
Source: BMC Health Serv Res. 2026 Nov 6;26:1558. doi: 10.1186/s12913-026-16041-5 (PMC13750032; doi:10.1186/s12913-026-16041-5)
Supplement: Supplementary file 1 [file 12913_2026_16041_MOESM1_ESM.docx]

**Appendix 1: Search strategy**

A) **Original search strategy:** **Health Business Elite**

**Database:** Health Business Elite (via EBSCOhost)

**Search fields**

All fields

**Combined PICO-based search string**

("digital health"
 OR ehealth
 OR "e health"
 OR telemedicine
 OR "distance counseling"
 OR "distance counselling"
 OR "e visit*"
 OR "e-visit*"
 OR "e consultation*"
 OR "e-consultation*"
 OR "digital encounter*"
 OR "chat based communication"
 OR "text based communication"
 OR "video consultation*")

AND

("primary health care"
 OR "primary care"
 OR "general practice")

AND

("health care cost*"
 OR "healthcare cost*"
 OR "cost benefit analys*"
 OR "cost-benefit analys*"
 OR "cost effectiveness analys*"
 OR "cost-effectiveness analys*"
 OR "cost minimization analys*"
 OR "cost-minimization analys*"
 OR "cost utility analys*"
 OR "cost-utility analys*")

**Limits and Filters**

Date searched: 18 January 2025

Publication years: 2015–2025

Language: No restrictions applied

Population: No restrictions applied

**Search Results**

The final search yielded 24 records after application of all limits.

**Methodological note**

Although Health Business Elite does not use MeSH, it provides a controlled subject heading system. Consequently, the search strategy combined database-specific subject headings with free-text terms reflecting the same concepts as those used in the PubMed search.

B) **Original search strategy:** **MEDLINE via PubMed**

**Database:** PubMed

Intervention (digital care):

("Digital Health"[MeSH]
OR "digital health"[free text]
OR "eHealth"[free text]
OR "e health"[free text]
OR "Telemedicine"[MeSH]
OR telemedicine[free text]
OR "Distance Counseling"[MeSH]
OR "distance counselling"[free text]
OR "e-visit*"[free text]
OR "e visit*"[free text]
OR "e-consultation*"[free text]
OR "e consultation*"[free text]
OR "digital encounter*"[free text]
OR "chat based communication"[free text]
OR "text based communication"[free text]
OR "video consultation*"[free text])

Control (traditional care):

("Primary Health Care"[MeSH]
OR "primary health care"[free text]
OR "primary care"[free text]
OR "General Practice"[MeSH]
OR "general practice"[free text])

Outcome (economic outcomes):

("Health Care Costs"[MeSH]
OR "health care cost*"[free text]
OR "healthcare cost*"[free text]
OR "Cost-Benefit Analysis"[MeSH]
OR "cost-benefit analys*"[free text]
OR "cost benefit analys*"[free text]
OR "Cost-Effectiveness Analysis"[MeSH]
OR "cost-effectiveness analys*"[free text]
OR "cost effective analys*"[free text]
OR "cost-minimization analys*"[free text]
OR "cost minimization analys*"[free text]
OR "cost-utility analys*"[free text]
OR "cost utility analys*"[free text]

**Combined PICO Search String**

The above three blocks were combined using Boolean AND operators in PubMed.

**Limits and Filters**

Date searched: 18 January 2025

Publication years: 2015–2025

Study types: Clinical Study, Clinical Trial, Controlled Clinical Trial, Observational Study, Randomized Controlled Trial

Language: No restrictions applied

Population: No restrictions applied

**Search Results**

The final search yielded 1,143 records after application of all limits.

C) **Original search strategy: Scopus**

**Database:** Scopus

**Search fields**

All fields

**Combined PICO-based search string**

("digital health"
 OR “ehealth”
 OR "e health"
 OR telemedicine
 OR "distance counseling"
 OR "distance counselling"
 OR "e visit*"
 OR "e-visit*"
 OR "e consultation*"
 OR "e-consultation*"
 OR "digital encounter*"
 OR "chat based communication"
 OR "text based communication"
 OR "video consultation*")

AND

("primary health care"
 OR "primary care"
 OR "general practice")

AND

("health care cost*"
 OR "healthcare cost*"
 OR "cost benefit analys*"
 OR "cost-benefit analys*"
 OR "cost effectiveness analys*"
 OR "cost-effectiveness analys*"
 OR "cost minimization analys*"
 OR "cost-minimization analys*"
 OR "cost utility analys*"
 OR "cost-utility analys*")

**Limits and Filters**

Date searched: 18 January 2025

Publication years: 2015–2025

Language: No restrictions applied

Population: No restrictions applied

**Search Results**

The final search yielded 3,725 records after application of the publication year limits.

**Methodological note**

Scopus does not use controlled vocabulary such as MeSH. Therefore, the same concepts identified in the PubMed search strategy were searched as free-text terms.

D) **Updated search strategy (January 2025 to September 2026): Health Business Elite**

The searches were updated in September 2026 using the same databases and search concepts as in the original search. The updated searches were restricted to records published from January 2025 to September 2026. The complete search strategies used for the update are presented below.

**Database:** Health Business Elite (via EBSCOhost)

**Search fields**

All fields

**Combined PICO-based search string**

("digital health"
 OR ehealth
 OR "e health"
 OR telemedicine
 OR "distance counseling"
 OR "distance counselling"
 OR "e visit*"
 OR "e-visit*"
 OR "e consultation*"
 OR "e-consultation*"
 OR "digital encounter*"
 OR "chat based communication"
 OR "text based communication"
 OR "video consultation*")

AND

("primary health care"
 OR "primary care"
 OR "general practice")

AND

("health care cost*"
 OR "healthcare cost*"
 OR "cost benefit analys*"
 OR "cost-benefit analys*"
 OR "cost effectiveness analys*"
 OR "cost-effectiveness analys*"
 OR "cost minimization analys*"
 OR "cost-minimization analys*"
 OR "cost utility analys*"
 OR "cost-utility analys*")

**Limits and Filters**

Date searched: 17 September 2026

Publication years: 2025–2026

Language: No restrictions applied

Population: No restrictions applied

**Search Results**

The final search yielded 9 records after application of all limits.

**Methodological note**

Although Health Business Elite does not use MeSH, it provides a controlled subject heading system. Consequently, the search strategy combined database-specific subject headings with free-text terms reflecting the same concepts as those used in the PubMed search.

E) **Updated search strategy (January 2025 to September 2026): MEDLINE via PubMed**

**Database:** PubMed

Intervention (digital care):

("Digital Health"[MeSH]
OR "digital health"[free text]
OR "eHealth"[free text]
OR "e health"[free text]
OR "Telemedicine"[MeSH]
OR telemedicine[free text]
OR "Distance Counseling"[MeSH]
OR "distance counselling"[free text]
OR "e-visit*"[free text]
OR "e visit*"[free text]
OR "e-consultation*"[free text]
OR "e consultation*"[free text]
OR "digital encounter*"[free text]
OR "chat based communication"[free text]
OR "text based communication"[free text]
OR "video consultation*"[free text])

Control (traditional care):

("Primary Health Care"[MeSH]
OR "primary health care"[free text]
OR "primary care"[free text]
OR "General Practice"[MeSH]
OR "general practice"[free text])

Outcome (economic outcomes):

("Health Care Costs"[MeSH]
OR "health care cost*"[free text]
OR "healthcare cost*"[free text]
OR "Cost-Benefit Analysis"[MeSH]
OR "cost-benefit analys*"[free text]
OR "cost benefit analys*"[free text]
OR "Cost-Effectiveness Analysis"[MeSH]
OR "cost-effectiveness analys*"[free text]
OR "cost effective analys*"[free text]
OR "cost-minimization analys*"[free text]
OR "cost minimization analys*"[free text]
OR "cost-utility analys*"[free text]
OR "cost utility analys*"[free text]

**Combined PICO Search String**

The above three blocks were combined using Boolean AND operators in PubMed.

**Limits and Filters**

Date searched: 17 September 2026

Publication years: 2025–2026

Study types: Clinical Study, Clinical Trial, Controlled Clinical Trial, Observational Study, Randomized Controlled Trial

Language: No restrictions applied

Population: No restrictions applied

**Search Results**

The final search yielded 546 records after application of all limits.

F) **Updated search strategy (January 2025 to September 2026): Scopus**

**Database:** Scopus

**Search fields**

Title-abstract-keyword

**Combined PICO-based search string**

("digital health"
 OR “ehealth”
 OR "e health"
 OR telemedicine
 OR "distance counseling"
 OR "distance counselling"
 OR "e visit*"
 OR "e-visit*"
 OR "e consultation*"
 OR "e-consultation*"
 OR "digital encounter*"
 OR "chat based communication"
 OR "text based communication"
 OR "video consultation*")

AND

("primary health care"
 OR "primary care"
 OR "general practice")

AND

("health care cost*"
 OR "healthcare cost*"
 OR "cost benefit analys*"
 OR "cost-benefit analys*"
 OR "cost effectiveness analys*"
 OR "cost-effectiveness analys*"
 OR "cost minimization analys*"
 OR "cost-minimization analys*"
 OR "cost utility analys*"
 OR "cost-utility analys*")

**Limits and Filters**

Date searched: 17 September 2026

Publication years: 2025–2026

Language: No restrictions applied

Population: No restrictions applied

**Search Results**

The final search yielded 103records after application of the publication year limits.

**Methodological note**

Scopus does not use controlled vocabulary such as MeSH. Therefore, the same concepts identified in the PubMed search strategy were searched as free-text terms.
