# Supplementary material for: Cost-effectiveness of digital health in primary care: a systematic review
Source: BMC Health Serv Res. 2026 Nov 6;26:1558. doi: 10.1186/s12913-026-16041-5 (PMC13750032; doi:10.1186/s12913-026-16041-5)
Supplement: Supplementary file 2 [file 12913_2026_16041_MOESM2_ESM.docx]

**Data extraction sheet**

Author

Year

Country

Reference

Population/disease

Intervention

Comparison

Occupational group

Study design

Perspective

Sample size

Differences in costs (95% CI)

Differences in effect (95% CI)

ICER (95% CI)

Net monetary benefit

Use of WTP/threshold (yes/no)

Currency and year

Cost of intervention

Full/partial evaluation

Analysis type (CEA/CBA/CUA/CA/CCA/DID)

Time horizon

Conclusions
