# Supplementary material for: Cost-effectiveness of digital health in primary care: a systematic review
Source: BMC Health Serv Res. 2026 Nov 6;26:1558. doi: 10.1186/s12913-026-16041-5 (PMC13750032; doi:10.1186/s12913-026-16041-5)
Supplement: Supplementary file 3 [file 12913_2026_16041_MOESM3_ESM.docx]

**JBI critical appraisal instrument, methodological quality assessment (n=54).**

|  | Q1 | Q2 | Q3 | Q4 | Q5 | Q6 | Q7 | Q8 | Q9 | Q10 | Q11 | Quality of Study |
| --- | --- | --- | --- | --- | --- | --- | --- | --- | --- | --- | --- | --- |
| **Partial Economic Evaluation** | | | | | | | | | | | | |
| CHEN et al. | Y | U | Y | U | Y | Y | Y | N | U | U | U | 5/11 |
| DAHLBERG et al. | Y | Y | N | N | Y | Y | Na | Na | Y | U | U | 5/11 |
| DE QUEIROZ PADILHA et al. | Y | Y | U | U | Y | Y | Y | N | N | Y | U | 6/11 |
| EKMAN | Y | Y | Y | N | Y | Y | Na | N | U | U | U | 5/11 |
| MALIK et al. | Y | Y | Y | N | U | U | Na | Y | Y | N | N | 5/11 |
| SAMPSON et al. | Y | Na | N | Y | Y | Y | N | N | Y | U | Y | 6/11 |
| WINWARD et al. | Y | Y | U | U | Y | Y | Y | N | N | U | Y | 6/11 |
| ZALESKI et al. | Y | Y | U | N | Y | Y | Na | U | U | U | U | 4/11 |
| **Full Economic Evaluation** | | | | | | | | | | | | |
| ABI HANA et al | Y | Y | Y | Y | Y | Y | Na | Y | Y | U | N | 8/11 |
| AXELSSON et al. | Y | Y | Y | Y | Y | U | Na | Y | Y | U | Y | 8/11 |
| BISSON et al. | Y | Y | Y | Y | Y | Y | Y | Y | Y | Y | Y | 11/11 |
| BROUWERS et al. | Y | Y | Y | Y | Y | Y | Na | Y | Y | Y | Y | 10/11 |
| BUNTROCK et al. | Y | Y | Y | Y | Y | Y | Na | Y | Y | Y | Y | 10/11 |
| CHUNG et al. | Y | Y | Y | Y | Y | Y | Na | Y | N | Y | U | 8/11 |
| CRUVINEL-JÚNIOR et al. | Y | Y | Y | Y | Y | Y | Na | Y | Y | Y | U | 9/11 |
| DE QUEIROZ PADILHA et al. | Y | Y | U | U | Y | Y | Y | N | N | Y | U | 6/11 |
| DIXON et al. (a) | Y | Y | Y | Y | Y | Y | Na | Y | Y | Y | Y | 10/11 |
| DIXON et al. (b) | Y | Y | Y | Y | Y | Y | Na | Y | Y | Y | Y | 10/11 |
| EVERITT et al. | Y | Y | Y | Y | Y | Y | Na | Y | Y | Y | Y | 10/11 |
| FALEH ALMUTAIRI et al. | Y | Y | Y | Y | U | Y | Na | Y | Y | Y | U | 8/11 |
| FATOYE et al. | Y | Y | U | Y | U | U | Na | Y | U | Y | U | 5/11 |
| FREDERIX et al. | Y | Y | Y | Y | Y | Y | N | Y | N | Y | U | 8/11 |
| FREITAG et al. | Y | Y | Y | Y | Y | Y | Y | Y | Y | U | Y | 10/11 |
| GERAGHTY et al. | Y | Y | Y | Y | Y | Y | Na | Y | Y | Y | Y | 10/11 |
| GREVING et al. | Y | Y | Y | Y | Y | Y | Na | Y | Y | Y | Y | 10/11 |
| HARVEY et al. | Y | Y | Y | Y | U | Y | Y | Y | Y | Y | U | 9/11 |
| HERMANS et al. | Y | Y | Y | Y | Y | Y | Na | Y | Y | Y | Y | 10/11 |
| KEBEDE et al. | Y | Y | Y | Y | Y | Y | Na | Y | Y | U | Y | 9/11 |
| KIM et al. | Y | Y | Y | Y | U | Y | Y | Y | N | U | U | 7/11 |
| KLOEK et al. | Y | Y | Y | Y | Y | Y | Na | Y | Y | Y | Y | 10/11 |
| KONGSTAD et al. | Y | Y | Y | Y | Y | Y | Na | Y | Y | Y | Y | 10/11 |
| KOPPENAAL et al. | Y | Y | Y | Y | Y | Y | Na | Y | Y | Y | Y | 10/11 |
| KUMAR et al. | Y | Y | Y | Y | Y | Y | Y | Y | Y | U | Y | 10/11 |
| LEE et al. (a) | Y | Y | Y | Y | Y | Y | Na | Y | Y | Y | Y | 10/11 |
| LEE et al. (b) | Y | Y | Y | Y | Y | Y | Na | Y | Y | Y | Y | 10/11 |
| LEWKOWICZ et al. | Y | Y | Y | Y | Y | Y | Y | Y | Y | Y | Y | 11/11 |
| LITTLE et al. | Y | Y | Y | Y | Y | Y | Na | Y | Y | Y | Y | 10/11 |
| LOOHUIS et al. | Y | Y | Y | Y | Y | Y | Y | Y | Y | U | Y | 10/11 |
| MICHAUD et al. | Y | Y | Y | Y | Y | Y | Na | Y | Y | Y | Y | 10/11 |
| MORRISS et al. | Y | Y | Y | Y | Y | Y | Na | Y | U | Y | Y | 9/11 |
| MUELLER et al. | Y | Y | Y | Y | Y | Y | Na | Y | Y | U | U | 8/11 |
| NORDYKE et al. | Y | Y | Y | Y | Y | U | Y | Y | Y | Y | Y | 10/11 |
| PARK et al. | Y | Y | Y | Y | Y | Y | Y | Y | Y | U | Y | 10/11 |
| PARKER et al. | Y | Y | U | Y | U | U | Na | N | Y | U | U | 4/11 |
| PELLE et al. | Y | Y | Y | Y | Y | Y | Na | Y | Y | Y | U | 9/11 |
| PRIEBE et al. | Y | Y | U | Y | Y | Y | Na | Y | N | Y | Y | 8/11 |
| ROMERO-SANCHIZ et al. | Y | Y | Y | Y | Y | Y | Na | Y | U | Y | Y | 9/11 |
| SJÖSTRÖM et al. | Y | Y | Y | Y | Y | Y | Na | Y | Y | Y | U | 9/11 |
| SMITH et al. | Y | Y | Y | Y | Y | U | Y | Y | Y | Y | Y | 10/11 |
| SOLOMON et al. | Y | Y | Y | Y | Y | Y | Na | Y | Y | Y | Y | 10/11 |
| TEIXEIRA et al. | Y | Y | Y | Y | Y | Y | Na | Y | Y | Y | Y | 10/11 |
| TOONDERS et al. | Y | Y | Y | Y | Y | Y | Na | Y | Y | Y | Y | 10/11 |
| YOU et al. | Y | Y | Y | Y | Y | Y | Y | Y | Y | Y | U | 10/11 |
| VOMHOF et al. | Y | Y | Y | Y | Y | Y | Na | Y | Y | Y | U | 9/11 |
| WALLER et al. | Y | Y | Y | Y | Y | Y | Na | Y | Y | Y | U | 9/11 |
| **Total %** | 100 | 96 | 85 | 87 | 89 | 89 | 26 | 85 | 78 | 70 | 61 |  |
|  | 54/54 | 52/54 | 46/54 | 47/54 | 48/54 | 48/54 | 14/54 | 46/54 | 42/54 | 38/54 | 33/54 |  |

*Y = Yes; N = No; Na= Not applicable; U = Unclear

JBI critical appraisal checklist for economic evaluations:

Q1 = Is there a well-defined question?

Q2 = Is there comprehensive description of alternatives?

Q3 = Are all important and relevant costs and outcomes for each alternative identified?

Q4 = Has clinical effectiveness been established?

Q5 = Are costs and outcomes measured accurately?

Q6 = Are costs and outcomes valued credibly?

Q7 = Are costs and outcomes adjusted for differential timing?

Q8 = Is there an incremental analysis of costs and consequences?

Q9 = Were sensitivity analyses conducted to investigate uncertainty in estimates of cost or consequences?

Q10 = Do study results include all issues of concern to users?

Q11 = Are the results generalizable to the setting of interest in the review
