# Supplementary material for: Cost-effectiveness of digital health in primary care: a systematic review
Source: BMC Health Serv Res. 2026 Nov 6;26:1558. doi: 10.1186/s12913-026-16041-5 (PMC13750032; doi:10.1186/s12913-026-16041-5)
Supplement: Supplementary file 4 [file 12913_2026_16041_MOESM4_ESM.docx]

**List of studies excluded at full text**

| **Author** | **Reason for exclusion** |
| --- | --- |
| EKERSUND, J., SAMUELSSON, E., LINDHOLM, L. & SJÖSTRÖM, M. 2022. | Control out of scope |
| JONES, G., BRENNAN, V., JACQUES, R., WOOD, H., DIXON, S. & RADLEY, S. 2018 | Population out of scope |
| MARINA, N., BAYÓN, J. C., LÓPEZ DE SANTA MARÍA, E., GUTIÉRREZ, A., INCHAUSTI, M., BUSTAMANTE, V. & GÁLDIZ, J. B. 2016. | Intervention out of scope |
| YU, J., HUCKFELDT, P. J., MINK, P. J., MEHROTRA, A. & ABRAHAM, J. M. 2023 | Outcome out of scope |
| KOUSKOUKIS, M. N. & BOTSARIS, C. 2017. | Intervention out of scope |
| WILLEMSEN, R. F., AARDOOM, J. J., VAN DER GALIËN, O. P., VAN DE VIJVER, S., CHAVANNES, N. H. & VERSLUIS, A. 2024. | Intervention out of scope |
| DEAR, B. F., KARIN, E., FOGLIATI, R., DUDENEY, J., NIELSSEN, O., SCOTT, A. J., GANDY, M., BISBY, M. A., HERISEANU, A. I., HATHWAY, T., STAPLES, L., TITOV, N. & SCHROEDER, L. 2021 | Control out of scope |
| FATOYE, F., GEBRYE, T., MBADA, C. E., FATOYE, C. T., MAKINDE, M. O., AYOMIDE, S. & IGE, B. 2022 | Intervention out of scope |
| FRITZ, J. M., MINICK, K. I., BRENNAN, G. P., MCGEE, T., LANE, E., SKOLASKY, R. L., THACKERAY, A., BARDSLEY, T., WEGENER, S. T. & HUNTER, S. J. 2022 | Outcome out of scope |
| GYLLENSTEN, H., LARSSON, A., BERGENHEIM, A., BARENFELD, E. & MANNERKORPI, K. 2024. | Intervention out of scope |
| LEWKOWICZ, D., BOTTINGER, E. & SIEGEL, M. 2023. | Included in another study |
| RHON, D. I., MAYHEW, R. J., GREENLEE, T. A. & FRITZ, J. M. 2021. | Outcome out of scope |
| SUMAN, A., SCHAAFSMA, F. G., VAN DONGEN, J. M., ELDERS, P. J. M., BUCHBINDER, R., VAN TULDER, M. W. & ANEMA, J. R. 2019. | Control out of scope |
| HARRIS, A., HINMAN, R. S., LAWFORD, B. J., EGERTON, T., KEATING, C., BROWN, C., METCALF, B., SPIERS, L., SUMITHRAN, P., QUICKE, J. G. & BENNELL, K. L. 2023 | Control out of scope |
| ROLLO, M. E., BURROWS, T., VINCZE, L. J., HARVEY, J., COLLINS, C. E. & HUTCHESSON, M. J. 2018. | Study type out of scope |
| BRABYN, S., ARAYA, R., BARKHAM, M., BOWER, P., COOPER, C., DUARTE, A., KESSLER, D., KNOWLES, S., LOVELL, K., LITTLEWOOD, E., MATTOCK, R., PALMER, S., PERVIN, J., RICHARDS, D., TALLON, D., WHITE, D., WALKER, S., WORTHY, G. & GILBODY, S. 2016 | Control out of scope |
| DEAR, B. F., ZOU, J. B., ALI, S., LORIAN, C. N., JOHNSTON, L., SHEEHAN, J., STAPLES, L. G., GANDY, M., FOGLIATI, V. J., KLEIN, B. & TITOV, N. 2015. | Control out of scope |
| EGEDE, L. E., DISMUKE, C. E., WALKER, R. J., ACIERNO, R. & FRUEH, B. C. 2018 | Population out of scope |
| EGEDE, L. E., GEBREGZIABHER, M., WALKER, R. J., PAYNE, E. H., ACIERNO, R. & FRUEH, B. C. 2017. | Population out of scope |
| LE, L. K. D., SANCI, L., CHATTERTON, M. L., KAUER, S., BUHAGIAR, K. & MIHALOPOULOS, C. 2019. | Intervention out of scope |
| RICHARDS, D., ENRIQUE, A., EILERT, N., FRANKLIN, M., PALACIOS, J., DUFFY, D., EARLEY, C., CHAPMAN, J., JELL, G., SOLLESSE, S. & TIMULAK, L. 2020 | Control out of scope |
| TITOV, N., DEAR, B. F., ALI, S., ZOU, J. B., LORIAN, C. N., JOHNSTON, L., TERIDES, M. D., KAYROUZ, R., KLEIN, B., GANDY, M. & FOGLIATI, V. J. 2015. | Control out of scope |
| WILLEMSEN, R. F., VERSLUIS, A., AARDOOM, J. J., PETRUS, A. H. J., SILVEN, A. V., CHAVANNES, N. H. & VAN DIJKE, A. 2024 | Outcome out of scope |
| ALSHAMMARI, T. M., ALSUBAIT, S. I., ALENZI, K. A. & ALMALKI, Z. S. 2023. | Intervention out of scope |
| ANGELL, B., LUNG, T., PRAVEEN, D., MAHARANI, A., SUJARWOTO, S., PALAGYI, A., OCEANDY, D., TAMPUBOLON, G., PATEL, A. & JAN, S. 2021 | Intervention out of scope |
| AVERY, G., COOK, D. & TALENS, S. 2016. | Intervention out of scope |
| BERNARD, L., VALSECCHI, V., MURA, T., AOUINTI, S., PADERN, G., FERREIRA, R., PASTOR, J., JORGENSEN, C., MERCIER, G. & PERS, Y. M. 2022 | Intervention out of scope |
| BOHINGAMU MUDIYANSELAGE, S., STEVENS, J., WATTS, J. J., TOSCANO, J., KOTOWICZ, M. A., STEINFORT, C. L., BELL, J., BYRNES, J., BRUCE, S., CARTER, S., HUNTER, C., BARRAND, C. & HAYLES, R. 2019. | Intervention out of scope |
| CARTER, H. E., WALLIS, S., MCGOWAN, K., GRAVES, N., PITT, R., COFFEY, S., PHILLIPS, R. & PARCELL, M. 2023 | Intervention out of scope |
| CHEN, F., SIEGO, C. V., JASIK, C. B., NORWOOD, T., BERESFORD, L., YANG, Z. & DALL, T. M. 2023. The Value of Virtual Physical Therapy for Musculoskeletal Care | Study type out of scope |
| CLARKE, M., FURSSE, J., BROWN-CONNOLLY, N. E., SHARMA, U. & JONES, R. 2018 | Intervention out of scope |
| CROWLEY, M. J., TARKINGTON, P. E., BOSWORTH, H. B., JEFFREYS, A. S., COFFMAN, C. J., MACIEJEWSKI, M. L., STEINHAUSER, K., SMITH, V. A., DAR, M. S., FREDRICKSON, S. K., MUNDY, A. C., STRAWBRIDGE, E. M., MARCANO, T. J., OVERBY, D. L., MAJETTE ELLIOTT, N. T., DANUS, S. & EDELMAN, D. 2022 | Control out of scope |
| DARKINS, A., KENDALL, S., EDMONSON, E., YOUNG, M. & STRESSEL, P. 2015. | Outcome out of scope |
| DE BATLLE, J., MASSIP, M., VARGIU, E., NADAL, N., FUENTES, A., BRAVO, M. O., MIRALLES, F., BARBE, F. & TORRES, G. 2021 | Intervention out of scope |
| EGEDE, L. E., DISMUKE, C. E., WALKER, R. J., WILLIAMS, J. S. & EILER, C. 2021. | Intervention out of scope |
| FRITZEN, K., BASINSKA, K., RUBIO-ALMANZA, M., NICOLUCCI, A., KENNON, B., VERGÈS, B., ZAKRZEWSKA, K. & SCHNELL, O. 2019 | Intervention out of scope |
| GILMER, T., BURGOS, J. L., CECILIA, M. & VARGAS-OJEDA, A. 2019 | Intervention out of scope |
| HAY, J. W., LEE, P. J., JIN, H., GUTERMAN, J. J., GROSS-SCHULMAN, S., ELL, K. & WU, S. 2018 | Intervention out of scope |
| HU, W., JOSEPH, S., LI, R., WOODS, E., SUN, J., SHEN, M., JAN, C. L., ZHU, Z., HE, M. & ZHANG, L. 2024. | Intervention out of scope |
| JIMÉNEZ-MARRERO, S., YUN, S., CAINZOS-ACHIRICA, M., ENJUANES, C., GARAY, A., FARRE, N., VERDÚ, J. M., LINAS, A., RUIZ, P., HIDALGO, E., CALERO, E. & COMÍN-COLET, J. 2020. | Outcome out of scope |
| KARIMI, N., SECHI, A. J., HARB, M., SAWYER, E., WILLIAMS, A. J., NG, W. & CONNOR, S. J. 2021 | Population out of scope |
| KARSLIOGLU FRENCH, E., KANTER, J., WINGER, M. E., WILLIAMS, K., GRUMSKI, T., SCHUSTER, J. & BECKJORD, E. 2023 | Population out of scope |
| LE GOFF-PRONOST, M., MOURGEON, B., BLANCHÈRE, J. P., TEOT, L., BENATEAU, H. & DOMPMARTIN, A. 2018. | Population out of scope |
| LEMINEN, A., TYKKYLÄINEN, M. & LAATIKAINEN, T. 2018 | Intervention out of scope |
| LI, J., PARROTT, S., SWEETING, M., FARMER, A., ROSS, J., DACK, C., PAL, K., YARDLEY, L., BARNARD, M., HUDDA, M., ALKHALDI, G. & MURRAY, E. 2018. | Control out of scope |
| LITTLE, P., STUART, B., HOBBS, F. R., KELLY, J., SMITH, E. R., BRADBURY, K. J., HUGHES, S., SMITH, P. W., MOORE, M. V., LEAN, M. E., MARGETTS, B. M., BYRNE, C. D., GRIFFIN, S., DAVOUDIANFAR, M., HOOPER, J., YAO, G., ZHU, S., RAFTERY, J. & YARDLEY, L. 2016. | Included in another study |
| LIU, T., ZHAN, Y., CHEN, S., ZHANG, W. & JIA, J. 2023. | Study type out of scope |
| LU, Z., LI, Y., HE, Y., ZHAI, Y., WU, J., WANG, J. & ZHAO, Z. 2021 | Outcome out of scope |
| MCCURRY, S. M., VON KORFF, M., MORIN, C. M., CUNNINGHAM, A., PIKE, K. C., THAKRAL, M., WELLMAN, R., YEUNG, K., ZHU, W. & VITIELLO, M. V. 2019 | Study type out of scope |
| MICHAUD, T. L., HILL, J. L., ESTABROOKS, P. A. & SU, D. 2023 | Intervention out of scope |
| MUDIYANSELAGE, S. B., STEVENS, J., TOSCANO, J., KOTOWICZ, M. A., STEINFORT, C. L., HAYLES, R. & WATTS, J. J. 2023 | Intervention out of scope |
| MUSTONEN, E., HÖRHAMMER, I., ABSETZ, P., PATJA, K., LAMMINTAKANEN, J., TALJA, M., KURONEN, R. & LINNA, M. 2020 | Intervention out of scope |
| NOBIS, S., EBERT, D. D., LEHR, D., SMIT, F., BUNTROCK, C., BERKING, M., BAUMEISTER, H., SNOEK, F., FUNK, B. & RIPER, H. 2018. | Control out of scope |
| OKSMAN, E., LINNA, M., HÖRHAMMER, I., LAMMINTAKANEN, J. & TALJA, M. 2017. | Intervention out of scope |
| PADILHA, F. V. Q., RODRIGUES, D. L. G., BELBER, G. S., MAEYAMA, M. A., SPINEL, L., PINHO, A., VITTI, A., OTERO, M. S., POMPERMAIER, G. B., DAMAS, T. B. & OLIVEIRA JUNIOR, H. 2024 | Intervention out of scope |
| PARK, S., WARD, T., SUDIMACK, A., COX, S. & BALLREICH, J. 2025 | Included in another study |
| PIERA-JIMÉNEZ, J., WINTERS, M., BROERS, E., VALERO-BOVER, D., HABIBOVIC, M., WIDDERSHOVEN, J. W. M. G., FOLKVORD, F. & LUPIÁÑEZ-VILLANUEVA, F. 2020. | Intervention out of scope |
| PORATH, A., IRONY, A., BOROBICK, A. S., NASSER, S., MALACHI, A., FUND, N. & KAUFMAN, G. 2017. | Intervention out of scope |
| SHAO, Y., STOECKER, C., HONG, D., NAUMAN, E., FONSECA, V., HU, G., BAZZANO, A. N., KABAGAMBE, E. K. & SHI, L. 2023 | Study type out of scope |
| SCHERRENBERG, M., ZEYMER, U., SCHNEIDER, S., VAN DER VELDE, A. E., WILHELM, M., VAN'T HOF, A. W. J., KOLKMAN, E., PRINS, L. F., PRESCOTT, E., ILIOU, M. C., PEÑA-GIL, C., ARDISSINO, D., DE KLUIVER, E. P. & DENDALE, P. 2021 | Control out of scope |
| SORIANO, J. B., GARCÍA-RÍO, F., VÁZQUEZ-ESPINOSA, E., CONFORTO, J. I., HERNANDO-SANZ, A., LÓPEZ-YEPES, L., GALERA-MARTÍNEZ, R., PECES-BARBA, G., GOTERA-RIVERA, C. M., PÉREZ-WARNISHER, M. T., SEGRELLES-CALVO, G., ZAMARRO, C., GONZÁLEZ-PONCE, P., RAMOS, M. I., JAFRI, S. & ANCOCHEA, J. 2018 | Intervention out of scope |
| STEN-GAHMBERG, S., PEDERSEN, K., HARSHEIM, I. G., LØYLAND, H. I., SNILSBERG, Ø., IVERSEN, T., GODAGER, G., SÆTHER, E. M. & ABELSEN, B. 2024. | Intervention out of scope |
| SYDOW, H., PRESCHER, S., KOEHLER, F., KOEHLER, K., DORENKAMP, M., SPETHMANN, S., WESTERHOFF, B., WAGNER, C. J., LIERSCH, S., REBSCHER, H., WOBBE-RIBINSKI, S., RINDFLEISCH, H., MÜLLER-RIEMENSCHNEIDER, F., WILLICH, S. N. & REINHOLD, T. 2022 | Intervention out of scope |
| TALBOOM-KAMP, E., KETELAAR, P. & VERSLUIS, A. 2021 | Intervention out of scope |
| TAYLOR, A. H., TAYLOR, R. S., INGRAM, W. M., ANOKYE, N., DEAN, S., JOLLY, K., MUTRIE, N., LAMBERT, J., YARDLEY, L., GREAVES, C., KING, J., MCADAM, C., STEELE, M., PRICE, L., STREETER, A., CHARLES, N., TERRY, R., WEBB, D., CAMPBELL, J., HUGHES, L., AINSWORTH, B., JONES, B., JANE, B., ERWIN, J., LITTLE, P., WOOLF, A. & CAVANAGH, C. 2020 | Intervention out of scope |
| UDSEN, F. W., LILHOLT, P. H., HEJLESEN, O. K. & EHLERS, L. H. 2017. | Intervention out of scope |
| VAN DIJK, S. E. M., POLS, A. D., ADRIAANSE, M. C., VAN MARWIJK, H. W. J., VAN TULDER, M. W. & BOSMANS, J. E. 2021 | Intervention out of scope |
| VESTERGAARD, A. S., HANSEN, L., SØRENSEN, S. S., JENSEN, M. B. & EHLERS, L. H. 2020. | Intervention out of scope |
| YANG, L., XU, J., KANG, C., BAI, Q., WANG, X., DU, S., ZHU, W. & WANG, J. 2022 | Intervention out of scope |
| ANTHONY, B., DOUNGSONG, K., MACLEOD, C., FLYNN, G., MASTERSON-ALGAR, P., GOULDEN, N., ET AL. 2025. | Intervention out of scope |
| FINKELSTEIN, E. A., GARDNER, D. S., THAM, K. W., GANDHI, M., CHEUNG, Y. B., BAIRAVI, J., ET AL. 2025. | Intervention out of scope |
| HUANG, Z., ZHOU, Z., MAI, Z., HE, Y., CHEN, B., ZHENG, C., ET AL. 2026. | Intervention out of scope |
| ROUKAS, C., MILLER, L., BÜTTNER, F. C., HAMBORG, T., GORDEEV, V. S., WILEMAN, V., ET AL. 2026. | Intervention out of scope |
| STEWART, C. J., BRAVATA, D. M., NELSON, M. T., DATTA, E. & BEHAL, R. 2025. | Population out of scope |
| WANG, Y., HUANG, L., WANG, R., XU, Z., MA, Y., YU, S., ET AL. 2026. | Population out of scope |
